# Genomic investigations of diverse corbiculate bee gut-associated Gilliamella reveal conserved pathways for energy metabolism, with diverse and variable energy sources

**DOI:** 10.1099/acmi.0.000793.v3

**Published:** 2024-08-15

**Authors:** Viet Hung Nguyen

**Affiliations:** 1 Project Genomes To Functional, Ecological, and Evolutionary Characterizations (Project G2FEEC), Ho Chi Minh City, Vietnam

**Keywords:** corbiculate bees, energy metabolism, fermentation, *Gilliamella*, respiration, symbionts

## Abstract

*Gilliamella* is a genus of bacteria commonly found as symbionts of corbiculate bees. Research into energy metabolism by this genus has predominantly been done through *in vivo* and *in vitro* experiments focused on the type species *Gilliamella apicola*. This study examined 95 publicly available genomes representing at least 18 *Gilliamella* species isolated predominantly from the hindgut of corbiculate bees. Energy metabolism pathways were found to be highly conserved across not only the *Gilliamella* but also other members of the family *Orbaceae*. Evidence suggests *Gilliamella* are capable of fermentation of both fumarate and pyruvate. Fermentation of the former produces succinate. Fermentation of the latter can produce acetate, ethanol, formate, and both isoforms of lactate for all *Gilliamella* and acetoin for some *G. apicola* strains. According to genomic evidence examined, all *Gilliamella* are only capable of respiration under microoxic conditions, while higher oxygen conditions likely inhibits respiration. Evidence suggests that the glycolysis and pentose phosphate pathways are essential mechanisms for the metabolism of energy sources, with the TCA cycle playing little to no role in energy metabolism for all *Gilliamella* species. Uptake of energy sources, i.e. sugars and derivatives, likely relies predominantly on the phospho*enol*-pyruvate-dependent phosphotransferase system. Differences in the utilized energy sources may confer fitness advantages associated with specific host species.

## Data Summary

The author declares no data deposition into repositories was required. The author confirms that all supporting data have been provided within the article or through supplementary data files.

## Introduction

Both honey bees and bumblebees contribute significantly to the pollination of plants both in the wild and in agriculture [1,3], and therefore, their welfare is of global concern. It is well known that the microbiota of these bees is highly conserved in healthy individuals, comprising a small number of core genera [4,5]. These symbionts are important in mediating host health [6,8] and have been the subject of extensive studies.

One of the genera commonly presented in the gut of healthy honey bees and bumblebees, specifically the ileum, is *Gilliamella* [9]. Research into energy metabolism patterns by *Gilliamella* so far suggests that members of this genus are strictly chemoorganotrophic [10,12] and can produce energy under anoxic and microoxic, but not fully oxic conditions [12,14]. Multiple studies have found evidence that *Gilliamella* are capable of fermentation, with products varying across strains, including formate, acetate, succinate and lactate [10,12]. However, the specific mechanisms underlying *Gilliamella* growth under microoxic conditions are still unclear. Ubiquinone-8 biosynthesis has been characterized by some but not all *Gilliamella* [12,14]. Examination of *Gilliamella* genomes did find some genes associated often with ubiquinone-8 biosynthesis, and many genes involved in respiration in general, to be lacking [13,15]. However, the resolution of such studies has not been enough to definitively characterize potential respiratory pathways nor if the differential patterns across strains may be phenotypic or genotypic.

Yet, studying the energy metabolism of *Gilliamella* is crucial to better understanding the ecology of these bacteria, specifically in determining their underlying factors mediating symbiosis. Utilization of different energy sources can potentially explain differential host colonization patterns. Meanwhile, examining the potential electron acceptors (i.e. oxic conditions) can be informative in determining how *Gilliamella* may survive outside of the host environment. Similarities in energy metabolism patterns across the genus and with other *Orbaceae* are essential in understanding the evolution of *Gilliamella* in general and co-evolution with honey bees and bumblebees in particular. Broadly, a better understanding of the underlying genomic pathways for energy metabolism would significantly contribute to better laboratory designs to culture these vital bee symbionts and answer essential questions about whether previously described patterns of energy metabolism may be phenotypic or genotypic. To expand on current knowledge of *Gilliamella* patterns of energy metabolism, I characterized publicly available *Gilliamella* genomes in the Integrated Microbial Genomes and Microbiomes (IMG/M) database [16] in the context of energy metabolism features and, where possible, in their functional, evolutionary and ecological implications.

## Methods

### Data acquisition and quality control

*Gilliamella* genomes were identified via the Advanced Search Builder available in the IMG/M web service [16], with ‘Gilliamella’ specified under the ‘Genus’ option of the ‘Taxonomy’ selector. Only genomes also existing in the National Center for Biotechnology Information (NCBI) database [17] were retained for further analysis to ensure NCBI taxonomic classifications were available. The genomes were loaded into a narrative in the KBase suite of bioinformatics tools v5.1.3 [18], and their completeness, contamination and strain heterogeneity were estimated with CheckM v1.0.18 [19]. Only genomes with <5 % contamination and no strain heterogeneity were retained for analysis. The contamination allowance was to reasonably account for potential gene duplication or horizontal genetic transfer (HGT) events. The strain heterogeneity restriction was to minimize the confounding effects of strain-level variations, so that wherever possible, broadly genus-wide conservation or disparities could be more clearly defined in this study.

### Characterization of energy metabolism

The retained *Gilliamella* genomes were examined for genes involved in energy metabolism based on pathways available in the MetaCyc [20] and Kyoto Encyclopaedia of Genes and Genomes (KEGG) [21] databases, along with peer-reviewed literature. This included genes involved in the uptake of sugars and derivatives that may be utilized as energy sources in substrate-level phosphorylation, such as via the glycolysis and fermentative pathways and in ATP synthesis via the electron transport chain (ETC), including membrane-bound enzymes and enzymatic complexes, electron donors, electron acceptors, electron-transfer quinones (ubiquinone, menaquinone, rhodoquinone, plastoquinone and phylloquinone) and electron-transfer c-type cytochromes. Where comparisons were made to other *Orbaceae*, this always involved all non-*Gilliamella* genomes existing in the IMG/M database (five *Frischella perrara*, two *Candidatus* Schmidhempelia bombi, one *Orbus hercynius* and one *Zophobihabitans entericus* (as of 24 April 2023, Table S1, available in the online version of this article).

### Phylogenetic analyses

The genomes of *O. hercynius* (GCF_003634275.1) and *F. perrara* (GCF_000807275.1) were additionally loaded into the aforementioned Kbase narrative to form the outgroup. The *Gilliamella* and outgroup genomes were combined into a Kbase GenomeSet by using ‘Batch Create Genome Set’ v1.2.0, and then a phylogenetic tree was built using 49 universally conserved Clusters of Orthologous Groups (COGs) identified from the 97 genomes using ‘Insert Set of Genomes into SpeciesTree’ v1.2.0. ‘Trim SpeciesTree to GenomeSet’ v1.4.0 was used to ensure the tree only contained the 97 (95 *Gilliamella* +2 outgroup) genomes of interest.

For all phylogenetic analyses of individual genes, description of homologs used and date acquired is mentioned in the respective figure legends. Multiple sequence alignment was performed with muscle (codon) using default parameters in mega X v10.0.5 [22] and then loaded into a narrative in KBase v5.1.3 [18], where the phylogenetic tree was constructed with FastTree v2.1.9 [23]. Trees were further processed via mega X or iTOL v6.8 [24] (specified in the respective figure legend) to add annotations and provide clearer visualization.

## Results and discussion

### Genome selection and metadata statistics

A total of 119 genomes classified to the genus *Gilliamella* were found in the IMG/M database (as of 24 February 2023). Of these genomes, 24 were removed due to estimated contamination of >5%, strain heterogeneity of >0 and/or data use restrictions, leaving 95 genomes retained for further analysis (Table S2). The estimated completeness of these 95 genomes ranges from 97.18 to 98.87%, although this is likely an underestimation as three CheckM marker genes (TIGR01347, TIGR00239 and PF03937) are missing from all genomes, suggesting this is a genomic trait of the genus rather than reflecting incomplete genome assembly. These three marker genes are involved in the TCA cycle, and further examination revealed three other genes core to the TCA cycle which were also missing from all 95 genomes, and almost all other genes were missing in 33. Estimated contamination for all 95 genomes ranges from 0 to 1.13%, suggesting little to no genomic impurity.

**Fig. 1.**
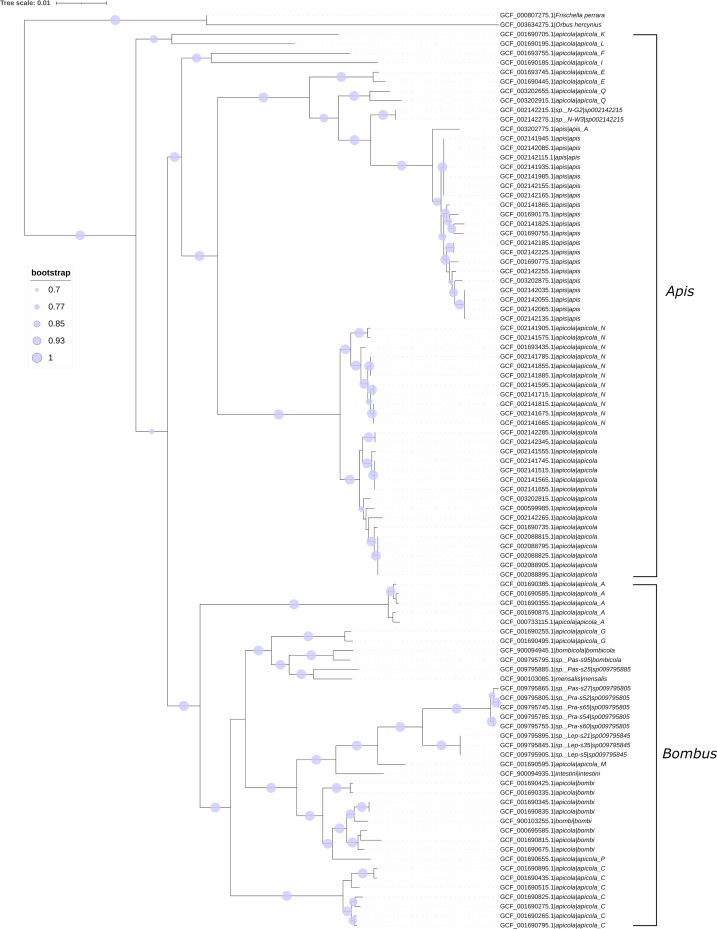
A phylogenetic tree of all 95 *Gilliamella* strains in this study based on 49 conserved COG domains, with the genomes of *O. hercynius* and *F. perrara* making the outgroup. RefSeq accessions are included for all 97 genomes. For *Gilliamella* genomes, only the species, but not genus, epithet is included to reduce redundancy. The first species epithet is per the NCBI database, and the second is per the Genome Taxonomy Database. Brackets represent the host genus of the *Gilliamella* clusters.

All 95 genomes were sequenced from corbiculate bee hosts, with 52 from *Apis mellifera* and 6 from 3 other *Apis* species, the remaining 43 genomes from 16 *Bombus* species. In 91 cases, the genomes were sequenced exclusively from the host gut, while 4 were from a mashup of the host gut and crop (Table S2). The genomes examined in this study, therefore, represent diverse species from diverse hosts, though not from diverse environments. The 95 genomes represent 18 species in the NCBI database (as of 24 February 2023) and 23 in the Genome Taxonomy Database (GTDB) Release 07-RS207 [25] (Table S2). The disparity is predominantly due to 47 genomes classified as *G. apicola* in the NCBI database but to other species in the GTDB. Phylogenetic analysis based on 49 conserved marker genes done in this study was more in line with the GTDB than the NCBI (Fig. 1). This includes a clear delineation between genomes sequenced from *Apis* versus *Bombus* hosts, which would be in line with the known degree of host-specificity and co-evolution of *Gilliamella* and corbiculate bees [9].

### Evidence for microaerophilic but not fully aerobic respiration

**Fig. 2.**
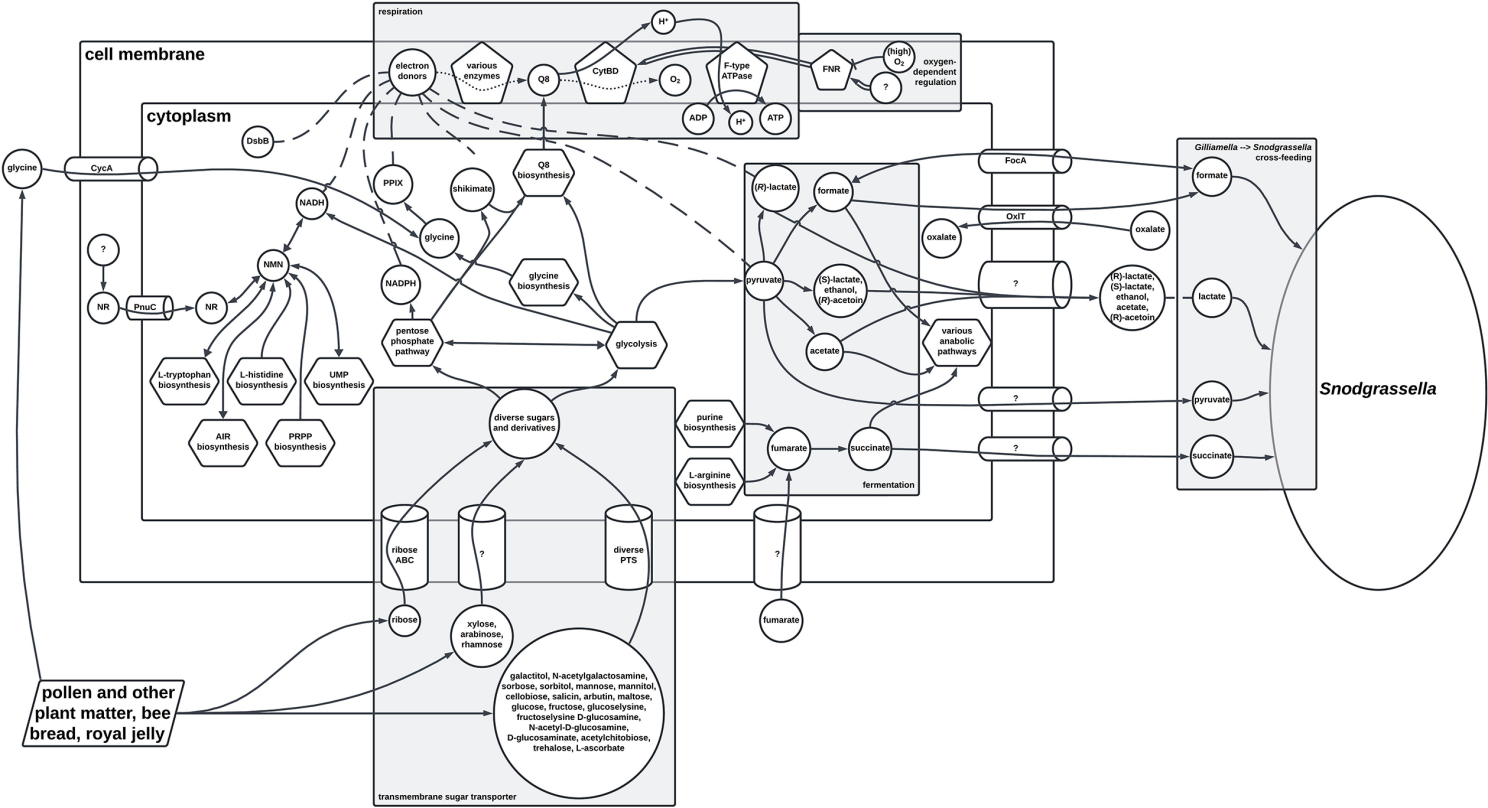
A simplified map showing the key features of the energy metabolism pathways of the genus *Gilliamella* based on genomic evidence analysed in this study, including potential cross-feeding of the co-occurring *Snodgrassella*. Pentagons represent enzymes, circles represent chemical(s), tubes represent transporter(s) or transport mechanisms, hexagons represent complete metabolic/biosynthesis pathways and trapezoids represent potential sources for certain compounds. A solid single arrow indicates a conversion between two different chemicals or transport between the same chemicals, a dashed single line indicates a chemical is a subset of a collection of multiple chemicals, a solid double arrow indicates activation and a solid single flat arrow indicates inhibition. A ‘?’ indicates unknown chemical(s) or transporter(s). For ease of visualization, chemicals undergoing similar metabolic or transport processes are clustered together. CytBD, cytochrome bd oxidase complex; F-type ATPase, F-type ATP synthase; FNR, fumarate and nitrate reduction regulatory protein (a global transcriptional regulator); Q8, ubiquinone-8/ubiquinol-8; ADP, adenosine diphosphate; ATP, adenosine triphosphate; NR, a nicotinamide riboside; NMN, a nicotinamide mononucleotide; NADH, reduced nicotinamide adenine dinucleotide; NADPH, reduced nicotinamide adenine dinucleotide phosphate; DsbB, protein dithiol:quinone oxidoreductase (disulfide-forming); PPIX, protoporphyrinogen IX; CycA, D-serine/D-alanine/glycine transporter; PnuC, nicotinamide riboside transporter; FocA, formate channel; OxlT, oxalate:formate antiporter; UMP, uridine 5′-monophosphate; AIR, 5-aminoimidazole ribonucleotide; PRPP, 5-phospho-α-d-ribose 1-diphosphate.

Respiration involves a redox reaction where electrons are transferred from an electron donor to a terminal electron acceptor. This process is facilitated by membrane-bound enzymes and/or enzymatic complexes, coupled with cation translocation across the cell membrane, to generate an electrochemical gradient that drives ATP synthesis via an F-type ATP synthase (ATPase) [26]. The presence of an F-type ATPase is, therefore, a strong indicator of the capacity for respiration, and with all eight possible genes encoding this complex [27] found in all 95 *Gilliamella* genomes examined, evidence suggests all members of this genus are capable of respiration, as summarized in Fig. 2.

In the ETC, the flow of electrons from an electron donor to the terminal electron acceptor often involves an intermediate electron-transfer compound, either quinones or c-type cytochromes. In all 95 genomes studied, I identified nearly all genes required for the biosynthesis of ubiquinone-8: those catalysing chorismate synthesis via the shikimate pathway [28], prenyl diphosphate and 3-methylbut-2-en-1-yl diphosphate via the methylerythritol pathway [29] and the subsequent anaerobic biosynthesis of ubiquinone-8 from these precursor compounds [30] (refer to Fig. S1). The only gene missing is *ubiC*, which aligns with previous research [13]. UbiC catalyses the conversion of chorismate to 4-hydroxybenzoate and in organisms like *Escherichia coli* is crucial for ubiquinone biosynthesis [31]. However, the 95 genomes examined include those of *Gilliamella* strains that have previously been found to produce ubiquinone-8 and, given the presence of all other genes necessary for ubiquinone-8 biosynthesis, suggests that all *Gilliamella* should nonetheless be capable of synthesizing this electron-transfer compound despite lacking *ubiC*. Other *Orbaceae* are also known to be capable of ubiquinone-8 biosynthesis [32,35], despite examination of their genomes also finding *ubiC* missing, further evidence that this gene is not necessary for ubiquinone-8 biosynthesis at least in this order. The likelihood that all *Gilliamella* are capable of ubiquinone-8 biosynthesis suggests that previous differences in ubiquinone-8 biosynthesis patterns [12,14] were due to phenotypic rather than genotypic factors. It is, however, unclear how chorismate conversion to 4-hydroxybenzoate occurs for *Gilliamella* as no other genes known to catalyse this reaction were found in any of the 95 genomes.

The gene *menA* involved in menaquinone biosynthesis could be found in 86 genomes. However, no other genes for this biosynthesis pathway could be identified, including for the precursor compound 1,4-dihydroxy-2-naphthoate [36]. This suggests that *menA* may rather play a different role or is non-functional for *Gilliamella*. No other genes involved in the biosynthesis of any other electron-transfer quinone could be identified, nor genes encoding electron-transfer cytochromes or enzymes/enzymatic complexes that utilize them. This is further evidence that biosynthesis of ubiquinone-8 must be possible for all *Gilliamella*, given it is the sole electron-transfer compound that can likely be produced.

Examination of all 95 *Gilliamella* genomes reveals only genes encoding a single membrane-bound enzymatic complex capable of utilizing an electron-transfer quinol as the electron donor, the cytochrome-bd oxidase complex (CytBD). CytBD utilizes oxygen as the terminal electron acceptor, though known to function exclusively under microoxic conditions [37]. No genes encoding any other ETC-linked enzyme or enzymatic complexes utilizing oxygen or any other substrate as the electron acceptor could be identified, evidence affirming that *Gilliamella* are indeed incapable of fully aerobic respiration as previously described [12,14]. Correspondingly, all genomes contain *fnr*, encoding the global transcriptional regulator induced during shifts between microoxic and anoxic conditions, but not *arcAB*, encoding the global transcriptional regulator activated during shifts between oxic and microoxic conditions [37]. The proposed inability to respire under oxic conditions suggests that although *Gilliamella* can be found outside of the host environment, commonly in pollen and other plant matter [38,39], such environments may rather act as limited reservoirs for horizontal transmission of *Gilliamella*, rather than habitats in which they are active in. It is possible that the potential disparity in activity and abundance within and outside the bee host influences the likelihood of horizontal transmission of these symbionts; thus, why research indicates the major route of acquisition of *Gilliamella* by honey bees is via social contact rather than from environmental sources [40].

The fumarate reductase is a membrane-bound enzymatic complex that allows fumarate to be utilized as a terminal electron acceptor in conjunction with an electron-transfer quinol as the electron donor. Thus, it is involved in anaerobic respiration. The archetypal fumarate reductase is encoded by four genes, *frdABCD*, with FrdAB involved in catalytic activity and FrdCD in membrane-anchoring [41]. Genes annotated as *frdA*, *frdC* and *frdD* by the IMG/M pipeline were identified in all 95 *Gilliamella* genomes. However, the gene annotated as *frdA* was located in a separate genomic region from *frdCD* in all cases, and a potential *frdA* homolog was only identified in the *Ca.* S. bombi Bimp and *Z. entericus* IPMB12 genomes, among other *Orbaceae* examined. The *Z. entericus* IPMB12 genome also contained *frdB*, with all four *frd* genes for this species located in the same genomic region syntenic to that containing only *frdCD* among all other *Orbaceae* examined, including *Gilliamella*. Further examination found *Gilliamella* and *Ca*. Schmidhempelia genes annotated as *frdA* to be ~900 bp, compared with ~600 bp of *Z. entericus* IPMB12, and the Enterobacterales and Pasteurellales, among other related taxa. The non-overlapping ~300 bp had no significant similarity to *frdB* or any other potential flanking genes, suggesting it did not represent a fusion of multiple genes. On the other hand, a BLASTp comparison to *frd* (encoding the ETC-independent, NADH-dependent fumarate reductase) in micro-organisms such as *Klebsiella pneumoniae* [42] found lower similarity but coverage across the entire ~900 bp of the gene, suggesting that the gene annotated as *frdA* in the *Gilliamella* and *Ca.* S. bombi Bimp genomes were rather mis-annotated by the IMG/M pipeline. Functional annotation with InterProScan 5 [43] confirmed that this gene should indeed rather be annotated as *frd*, rather than *frdA*. Phylogenetic analysis suggests that the *Gilliamella frd* may have been acquired via horizontal genetic transfer (Fig. S2A), given a lack of congruency with the currently proposed phylogeny [44]. In contrast, phylogenetic analysis of *Gillamella frdCD* suggests both genes were indeed acquired by descent (Fig. S2B and C), given the similarities to the currently known Gammaproteobacteria phylogeny [32]. This suggests that *frdAB* was likely lost after the split of other *Orbaceae* from the *Zophobihabitans*, and then later, *frd* was acquired prior to the split between the *Gilliamella* and *Candidatus* Schmidhempelia.

Although *frdCD* was retained in all *Orbaceae*, these genes are only known to encode the anchoring portion of the ETC-linked fumarate reductase [41], and therefore what roles they may still play, if any, is unclear. Similarly, all 95 *Gilliamella* genomes contain *nuoJKLMN*, 5 of the 14 *nuo* genes encode the archetypal proton-translocating NADH:ubiquinone oxidoreductase complex [45], consistent with previous findings for *G. apicola* [15]. Yet, while NuoLMN subunits are evidenced to be integral in catalysing proton translocation across the cellular membrane [46,48], none of the subunits encoded by *nuoJKLMN* are known to catalyse the oxidation of NADH. Despite the conserved nature of *frdCD* and *nuoJKLMN* across the *Orbaceae*, the lack of genes encoding important catalytic subunits rather suggests these genes may only be remnants of genomic reduction as previously hypothesized for *G. apicola* and *Ca.* S. bombi Bimp [15,32]. This may also similarly explain the lack of genes involved in menaquinone biosynthesis, as menaquinol is known to be the preferred electron-transfer quinol oxidized by the ETC-linked fumarate reductase complex [44].

All 95 *Gilliamella* genomes contain *ndh*, *hemG*, *dsbB*, *dld* and *nfsB*, 87 contain *poxB*, and 7 contain *quiA*. These seven genes encode seven different membrane-bound enzymes known to catalyse the reduction of electron-transfer quinones coupled with NADH, protoporphyrinogen IX, DsbA in its reduced form, (*R)-*lactate, NAD(P)H, pyruvate and shikimate, respectively, as electron donors [49,55] (Fig. S3). For some micro-organisms, QuiA can also catalyse the oxidation of l-quinate [54]. However, no genes necessary for quinate biosynthesis or transport could be identified from any of the seven genomes containing *quiA*, suggesting that the *Gilliamella* QuiA is likely only involved in shikimate utilization as an energy source.

Genetic evidence suggests *Gilliamella* can synthesize its electron donors directly via diverse metabolic pathways. Shikimate is an intermediate in chorismate biosynthesis [28], although, of note, genetic evidence suggests that this pathway is oxygen-dependent for *Gilliamella*. All 95 *Gilliamella* genomes contain genes encoding multiple pathways for NADH biosynthesis, either via 5-phospho-ɑ-d-ribose 1-diphosphate produced as a by-product of five different biosynthesis pathways or from 1-(β-D ribofuranosyl)nicotinamide that may be scavenged from the environment (Fig. S4). Pyruvate can be produced both via glycolysis and the pentose phosphate pathway, while (*R*)-lactate can be produced via fermentation, as described in later sections. Protoporphyrinogen IX, in particular, is often intermediate in the biosynthesis of either haem b or chlorophyll [56,57], and while genes were found for biosynthesis of this compound from glycine in all 95 genomes examined, no genes for further conversions could be identified, suggesting that *Gilliamella* may rather synthesize protoporphyrinogen IX exclusively for utilization as an electron donor in the ETC. Glycine has been found in pollen, royal jelly and bee bread [58], so feeding activities can result in the accumulation of glycine in the host gut (Fig. 2). Given all genomes also contain *cycA*, which encodes an effective glycine transporter [59], utilization of protoporphyrinogen IX as an electron donor may be more specifically a means to scavenge energy from glycine that may be readily available in the host environment.

The proposed capacity for microaerophilic, but not fully aerobic respiration (Fig. 2), is consistent with other symbionts that inhabit the gut of corbiculate bees [32,60, 61]. None of the ETC-linked enzymes or enzymatic complexes that the *Gilliamella* genomes encode can translocate cations across the cell membrane except for CytBD, and therefore, *Gilliamella* would have to rely solely on this complex to generate the cation motive force required for ATP synthesis. Nonetheless, the diverse possible electron donors suggest that microaerophilic respiration can occur efficiently for *Gilliamella* and potentially explain both their often abundance across the host environment [62] and the capacity to substitute other symbionts following dysbiosis [63], given the corbiculate bee inner environments can often be depleted of oxygen.

Based on the genomic features identified, there seems to be a disparity in oxic conditions for ubiquinone-8 biosynthesis and respiration by *Gilliamella*. Although *ubiI* was identified from all 95 *Gilliamella* genomes, no other genes involved in aerobic ubiquinone biosynthesis [30] were found. It is possible that ubiquinone-8 is indeed solely synthesized under anoxic conditions and may even play a role in regulating microaerophilic respiration. Alternatively, homologs of *ubi* genes have been found to catalyse alternative hydroxylation steps [30], and therefore, it may be of research interest to investigate whether the *Gilliamella ubiI* similarly catalyses alternative hydroxylation reactions, potentially allowing for *Gilliamella* to synthesize ubiquinone outside of anoxic conditions.

### Pathways for fermentation of pyruvate to acetate, lactate, ethanol and acetoin

Aside from evidence for fumarate fermentation to succinate, genes for pyruvate fermentation to lactate [both (*R)-* and (*S)-* isoforms], ethanol, formate and acetate were found in all genomes (Fig. S5). This suggests that *Gilliamella* are not only capable of fermentation via diverse pathways as previously identified [11], but that these pathways are conserved across the entire genus, and therefore differential fermentation patterns previously characterized [10,12] were due to phenotypic rather than genotypic factors. The exception is acetoin, with genes catalysing pyruvate fermentation to this compound found exclusively in 16 *G*. *apicola* genomes (Fig. S5). Fumarate fermentation often co-occurs with other fermentative pathways as part of mixed acid fermentation, with intermediates produced from glycolysis and via the reverse TCA cycle [64]. However, no genes necessary for fumarate synthesis from oxaloacetate via the reverse TCA cycle were found in any of the 95 *Gilliamella* genomes, suggesting that fumarate fermentation by *Gilliamella* is disconnected from the other fermentation pathways (Fig. S5). Instead, genes for the biosynthesis of fumarate as a byproduct of purine and l-arginine biosynthesis [65,66] were found in all 95 *Gilliamella* genomes, suggesting that fumarate fermentation by *Gilliamella* may rather be an auxiliary mechanism to scavenge energy from other metabolic processes. Although no genes encoding fumarate transporters could be identified from any of the 95 *Gilliamella* genomes, members of this genus are known to be able to import exogenous fumarate [67]. A novel, undescribed mechanism to import fumarate would be unsurprising, given that *Gilliamella* are known to be capable of importing other carboxylates also through a currently unknown mechanism [10].

Of note is that all *Gilliamella* genomes contain genes involved in three separate reactions converting pyruvate to acetyl-CoA, the first intermediate in the *Gilliamella* fermentation pathway to acetate/ethanol (Fig. S5). Each of these three reactions utilizes different substrates and yields different byproducts, with one forming formate and two forming carbon dioxide (Fig. S5). The redundancy suggests this fermentation pathway may be particularly favoured by *Gilliamella* and could explain why acetate was characterized as the dominant *Gilliamella* fermentation product, even across different experimental conditions [11]. This pathway is the sole fermentation pathway found for *Gilliamella* that yields ATP (Fig. S5) and has been suggested as the reason for its preference over other fermentation pathways in some other micro-organisms [68]. As this and the fermentation pathway to ethanol compete for the same intermediate (acetyl-CoA), the preference for fermentation to acetate may also explain the absence of ethanol detected despite the transcription of *adhE* identified [11].

### The phospho*enol*-pyruvate-dependent phosphotransferase system as the dominant mechanism for uptake of sugars and derivatives

One copy of *ptsI* and either two or three copies of *ptsH* could be identified in all 95 *Gilliamella* genomes examined. *PtsI* and *PtsH* together form the core, conserved component of the phospho*enol*-pyruvate-dependent phosphotransferase system (PTS), an inter-membrane transport system specifically of sugars and derivatives [69]. *PtsI* and *PtsH* catalyse the dephosphorylation of phosphoenol-pyruvate and subsequent phosphorylation of the transported substrate, respectively [69]. The PTS also comprises a variable component directly responsible for the translocation of the substrate across the cell membrane, the enzymatic makeup of this component controlling the substrate specificity of the system. An abundance of genes encoding this variable component was found across the 95 *Gilliamella* genomes, suggesting that *Gilliamella* are capable of synthesizing multiple variants of the PTS and therefore capable of uptaking diverse sugars and derivatives.

Specifically, all 95 *Gilliamella* genomes examined contain *crr*, *bglF*, *nagE* and *fruA*, each encoding a different variable component of the PTS responsible for the uptake of glucose, fructose, arbutin, salicin, cellobiose and glucosamine (both d-glucosamine and N-acetyl-d-Glucosamine) from the environment (Fig. S6), further suggesting that utilization of these substrates to likely be core to *Gilliamella* energy and/or carbon metabolism. Present in at least 76, but not all of the genomes, are genes that allow for the import of mannose, ascorbate, trehalose, galactitol, chitobiose and mannitol via the PTS, suggesting that the capacity to utilize these substrates to still be generally important, but not crucial for all *Gilliamella*. More auxiliary substrates that may be imported include galactosamine, sorbitol, sorbose, maltose, fructoselysine and glucoselysine, with genes encoding the variable PTS component specific for these substrates, found in 44 to 66 of the genomes (Fig. S6). All four *dgaABCD* genes, typically known to be responsible for the uptake of glucosaminate via the PTS, could be found in one genome, and *dgaC* could be found in 48 others. Yet, no existing study had found *Gilliamella* to be capable of degrading glucosaminate, and no genes involved in further glucosaminate metabolism could be found in any of the genomes in this study. This suggests that the *dga* genes identified may be non-functional or play a different role aside from glucosaminate transport and that *Gilliamella* are unable to produce a functional glucosaminate-specific PTS (Fig. S6).

**Fig. 3.**
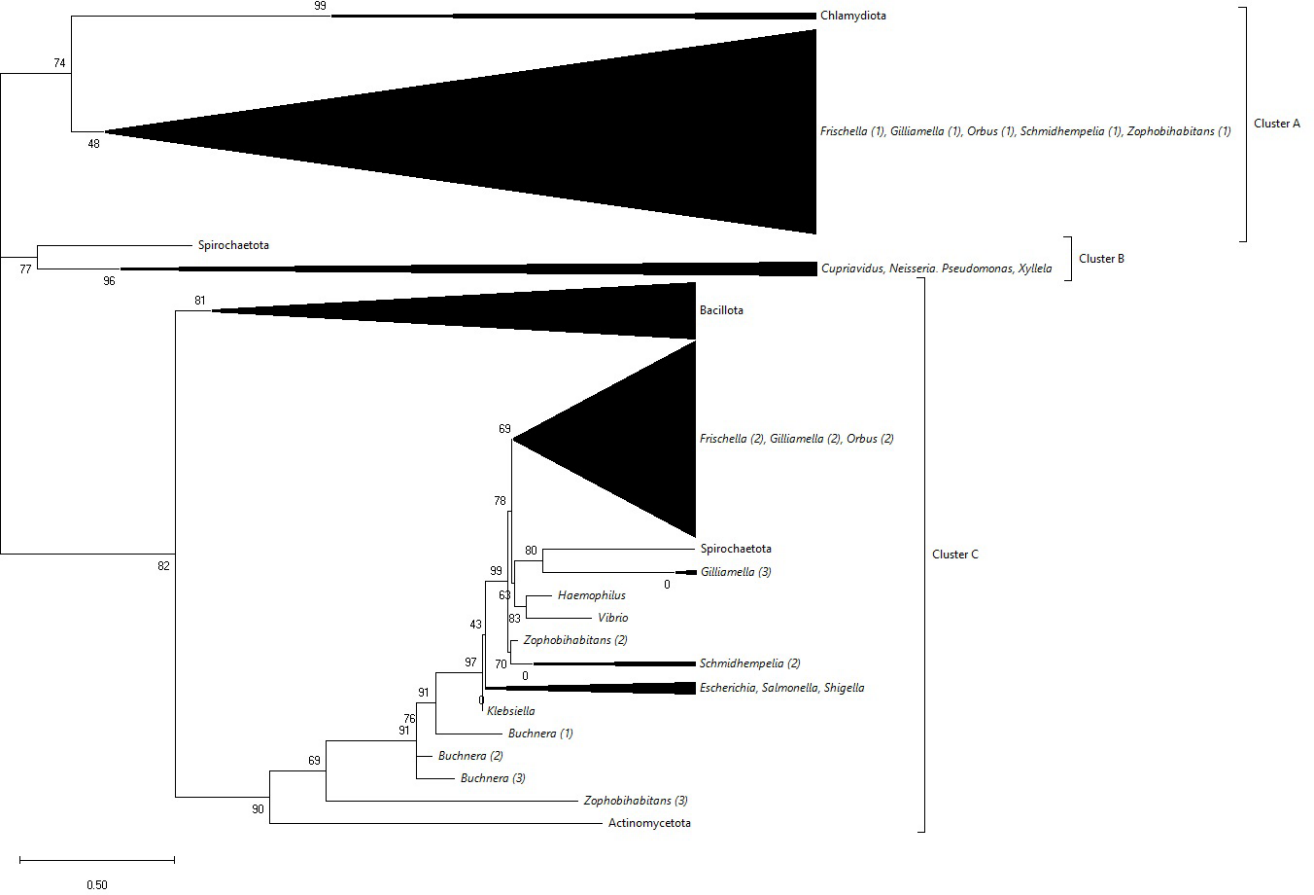
Unrooted phylogenetic tree of the *ptsH* homologs identified in all 95 *Gilliamella* genomes in this study and all other *Orbaceae* (as per Materials and Methods), along with all 57 reviewed (Swiss-Prot) reference homologs from the UniProtKB database (as of 26 March 2023). Numbers at the root of branches indicate bootstrap values. Branch length reflects divergence. Sequences derived from *Pseudomonadota* (formerly *Proteobacteria* [70]) were labelled down to the genus level, including all *Orbaceae*, while in all other cases were labelled at the phylum level. In cases where sequences from the same taxon formed distinct branches, numbering in brackets is included. Formatting and annotation was done via mega X.

For the two sets of *ptsH* homologs (here on *ptsH_A* and *ptsH_B*) found in every *Gilliamella* genome, the phylogenetic analysis found they were clustered with homologs from other *Orbaceae* (Fig. 3, Clusters A and C), and, therefore, both likely were already present early in the evolution of the family. However, according to the phylogenetic analysis, the closest known homologs to *ptsH_A* outside the *Orbaceae* were from *Chlamydiota* (Fig. 3, Cluster A). In contrast, *ptsH_B* formed a cluster with homologs from other *Gammaproteobacteria*, ignoring a single sequence from *Borreliaceae* (phylum *Spirochaetota*) (Fig. 3, Cluster C). Given the majority of *Gammaproteobacteria* only have one copy of the *ptsH* gene, this suggests that *ptsH_B* was acquired early in the evolution of the class, if not the phylum *Pseudomonadota* (formerly *Proteobacteria* [70]). In contrast, the distinctiveness of *ptsH_A* suggests it was acquired early in the evolution of the family *Orbaceae* via horizontal genetic transfer, although whether from *Chlamydiota* or otherwise is unclear given the phylogenetic distance (Fig. 3, Cluster A). The *ptsH_A* homologs shared ≥94.12%, while *ptsH_B* shared ≥94.25%, sequence identity, while the two homologs within each genome only shared <30% sequence identity, further evidence that the two homologs did not arise from a (relatively recent) duplication event.

The third *ptsH* homolog (*ptsH_C*) found in the genomes of *G. apicola* N-G5 and *G. apicola* ESL0182 shared 55.95 and 57.14% sequence identity to the *ptsH_B* homolog from the same genome. It should be noted that these two *Gilliamella* strains are phylogenetically distinct (Fig. 1, GCF_003202655.1 and GCF_003202655.1). It is, therefore, extremely unlikely that *ptsH_C* was lost in all *Orbaceae* except two distinct *Gilliamella* strains. In the *ptsH* phylogenetic tree, the two *Gilliamella ptsH_C* homologs also formed a distinct cluster with a homolog from *Spirochaetota* (Fig. 3, Cluster C). Together, this suggests a high likelihood that acquisition of *ptsH_C* by *G. apicola* N-G5 and *G. apicola* ESL0182 was via horizontal genetic transfer. However, there is not enough existing data to ascertain which exact taxon *G. apicola* N-G5 and *G. apicola* ESL0182 likely acquired *ptsH_C* from, nor whether it was two entirely independent events or if one *Gilliamella* strain passed the homolog to the other.

Despite the sequence dissimilarity between *ptsH_A* and *ptsH_B*, they nonetheless are functionally similar according to annotation via InterProScan5 [43]. While *ptsH_B* is found within the *pts* operon in all genomes, *ptsH_A* is found in a distinct region enriched with hypothetical proteins. The conservation of both homologs suggests they both play important roles. At this stage, there is however not enough data to elucidate the potential role of *ptsH_A*. Annotation by InterProScan5 [43] also suggests that *ptsH_C* is functionally similar to *ptsH_A* and *ptsH_B*. Both *ptsH_C* homologs are flanked upstream by a gene encoding a fructose-specific component of the PTS, along with other fructose metabolism genes. This potential fructose metabolism operon is distinct from a second variant also found within the genomes of these two strains and in all other *Gilliamella* examined. Further research is needed to determine if the additional fructose metabolism genes (and *ptsH_C*) play a redundant role or if they may actually enhance the fructose metabolism capacity of *G. apicola* N-G5 and *G. apicola* ESL0182.

Only genes encoding one other known mechanism of sugar import could be identified, specifically *rbs genes* in 60 of the 95 genomes (Fig. S6). In 40 of the 60 genomes, *rbsABCD* could be found within a possible operon with a LacI(-like) transcriptional regulator. In the other 20 genomes, all representing *G. apis*, *rbsABC* could be found in a potential operon with a DeoR/GlpR-like transcriptional regulator. This delineation suggests two distinct origins for these *rbs* genes in *Gilliamella*. Indeed, the *rbsABCD* + LacI(-like) transcriptional regulator operon is also found in the genome of *O. hercynius*, suggesting it was acquired early on in the evolution of the family and then lost in some *Gilliamella* taxa. On the other hand, the *rbsABC* +DeoR/GlpR-like transcriptional regulator operon is likely subsequently acquired exclusively by *G. apis*, potentially via HGT. There was one genome classified as *G. apis* ESL0172 in the NCBI database that contained the *rbsABCD* + LacI transcriptional regulator, rather than the *rbsABC* + DeoR/GlpR-like transcriptional regulator operon. However, given the exclusivity recognized in all other cases and that this same genome was classified to a different species than *G. apis* in the GTDB (Table S2), as does the phylogenetic analysis places it distinctly from all *G. apis* genomes (Fig. 1), it is likely that this genome was taxonomically misclassified in the NCBI database and that the *rbsABC* + DeoR/GlpR-like transcriptional regulator operon is truly unique to *G. apis* among the *Gilliamella*. How *G. apis* acquired these genes and from what source, along with any potential ecological roles, requires further research. RbsABC can comprise a fully functional ribose transporter [71], with RbsD an optional component [71,72], and hence all 60 *Gilliamella* strains are likely capable of producing a functional ribose transporter with their set of *rbsABC* or *rbsABCD* genes. Phylogenetic analysis of *rbs* genes supports this characterization, with *rbs* genes in either operon split across two distinct clusters, including the *rbs* homologs found for *G. apis* ESL0172 expectedly distinct from the remaining *G. apis rbs* homologs (Fig. S7A to S7C). Also expected, the *rbsD* homologs form a single cluster (Fig. S7D) with similar distributions to the *rbsABC* homologs in the same operon. Some *G. apicola* and all *G. apis* genomes also contain a second copy of *rbsB* and *rbsC*; they are not found in an operon with rbsA or rbsD and are flanked with a gene annotated to encode a fructokinase rather than a specific ribokinase and, therefore, do not seem to be involved in ribose transport. Expectedly, phylogenetic analysis placed these *rbsB* and *rbsC* homologs each in a third cluster (Fig. S7B and C).

Of the 95 genomes, 34, 54 and 57 contain genes necessary for arabinose (*araAB*), xylose (*xylAB*) and rhamnose (*rhaABD*) metabolism, respectively, but no genes encoding known transporters of these sugars – whether specific ABC or broad-spectrum carboxylate transporters. A previous study had found the same genomic patterns for *Gilliamella* strains that were found to be capable of uptake and metabolism of these carboxylates, suggesting *Gilliamella* employs a currently unknown transporter for these substrates [10]. Thus, although no genes encoding known transporters were found, it is still likely that the 34, 54 and 57 strains characterized with the respective genes for arabinose, xylose and rhamnose degradation should also similarly be able to uptake them from the environment.

The capacity to uptake diverse sugars and derivatives has previously been evident for *G. apicola* and a number of other *Gilliamella* species [10,12,15]. Here, evidence suggests the capacity to uptake diverse sugars and derivatives is not only widespread across the entire genus, but that the trait may have been acquired before the evolution of the genus itself, potentially as a pre-adaptation to the bee gut environment. Given the diverse plant-derived matters that bees may digest, it is possible that most if not all sugars *Gilliamella* may be capable of digesting may be derived from the host’s diet (Fig. 2). Although the PTS does seem to be the predominant mechanism employed by *Gilliamella* for the uptake of sugars and similar substrates, the likelihood of unknown carboxylate transporters does highlight that there may be even more exogenous substrates that can be utilized by *Gilliamella* than could currently be characterized, and, therefore, additional research into this aspect of *Gilliamella* biology would continue to be of interest.

Identification of genes necessary for both the import and metabolism of glucose and fructose across all genomes is evidence that previous differential patterns of utilization of these substrates [10] were phenotypic rather than genotypic. However, the mechanisms that regulate the uptake and metabolism of these and other sugars by *Gilliamella* are unclear. None of the 95 genomes contain *mlc*, which encodes a well-known transcriptional regulator specific to PTS [73], and while the presence of different sugars can regulate the transcription of different PTS variants [74], it nonetheless cannot explain a complete absence of sugar utilization [10]. Regulation of sugar uptake and metabolism by *Gilliamella*, therefore, also requires further investigations.

### The importance of the glycolysis and pentose phosphate pathways in connecting the various *Gilliamella* energy metabolism processes

An abundance of genes encoding the glycolysis and pentose phosphate pathways according to KEGG maps [21] were found in all 95 *Gilliamella* genomes examined. Specifically, 21 genes involved in glycolysis were identified in all 95 genomes, and a further 13 genes were identified in up to 91 genomes, while 15 genes involved in the pentose phosphate pathway were found in all genomes, and a further 16 were found in up to 89 genomes. This is unsurprising given the extent to which *Gilliamella* energy metabolism processes so far described are linked to these two pathways (Fig. 2). Genes were found for every strain to be able to metabolize the sugars and derivatives they are likely capable of uptaking, either directly via one or both of the two pathways or first via conversion to an intermediate compound, specifically glucose 6-phosphate, d-xylulose 5-phosphate, glycerone phosphate or β-d-glucose phosphate (Fig. S6). Pyruvate is the end product of glycolysis, which is not only the proposed substrate for all but one *Gilliamella* fermentation pathway (Fig. S5), but also a potential electron donor for ETC-linked microaerophilic respiration for at least 87 strains (Fig. S3). Biosynthesis of other components of the proposed *Gilliamella* ETC also occurs via these two pathways, specifically shikimate and ubiquinone-8. Lastly, the glycolysis and the pentose phosphate pathways are also important in, respectively, regenerating two potential electron donors for the ETC, NADH and NADPH, from their oxidized forms.

In contrast, genes encoding only a partial TCA cycle necessary for converting acetyl-CoA to 2-oxoglutarate could be identified from only 62 *Gilliamella* genomes, likely involved in catabolic processes such as amino acid biosynthesis rather than energy metabolism. Other genes in the TCA cycle found in all 95 genomes are also either involved in other catabolic pathways or, as aforementioned (i.e. *frdCD*), are likely remnants of genome reduction. This is significant as the TCA cycle is traditionally considered to play a more important role in regenerating NADH from NAD^+^ than glycolysis [75,76]. It is possible that the potential to produce NADH from diverse energy sources via glycolysis compensates for the lack of NADH produced via the TCA cycle, and therefore *Gilliamella* glycolysis plays all the more important role in energy metabolism.

### Fates of the energy metabolism end products

Of the potential products of fermentation, no genes could be found for the utilization of ethanol, either isoform of acetoin or (*S*)-lactate in any of the 95 *Gilliamella* genomes, suggesting that these compounds, if produced, are only exported to the external environment. As aforementioned, genetic evidence suggests all *Gilliamella* can utilize (*R*)-lactate; however, this would likely only be possible under microoxic conditions and may not be able to occur concurrently with fermentation. Conversely, the product of (*R*)-lactate oxidation via the ETC is pyruvate [77], which genetic evidence suggests it is the substrate utilized in all *Gilliamella* fermentation pathways, although only fermentation to acetate would yield additional ATP. It may be possible that this circular metabolism may be a mechanism to maximize resource usage, with (*R*)-lactate potentially uptaken from the environment and utilized as an electron donor under microoxic conditions, then for pyruvate to be utilized for fermentation, or vice versa with fermentation of pyruvate to (*R*)-lactate acting as a temporary energy sink until conditions turn microoxic, bypassing the requirement for NADH to act as a direct electron donor for the ETC. Genes were found in all 95 genomes necessary for the biosynthesis of the citrate lyase complex [78] specifically with the final acetylation step utilizing acetate, suggesting that acetate produced from fermentation (and potentially microaerophilic respiration using pyruvate as an electron donor for 87 strains (Fig. S3)) can be recycled for other metabolic processes, although the product of citrate degradation nonetheless yields more acetate [79]. The multiple processes that can yield acetate may further explain the higher presence of acetate than other fermentation products characterized [11].

All 95 genomes contain *focA*, encoding a bidirectional formate transporter [80], with the directionality regulated by the pH of the external environment [81]. The pH of the gut of corbiculate bees is known to be buffered by host mechanisms but could also be altered by activities of symbionts, including fermentation [82]. This suggests that formate transport by *Gilliamella* could be influenced not only by the host but also by other symbionts and even potentially self-regulated, given the ubiquity of fermentation capabilities characterized. Thirty-two of the genomes also contain *oxlT*, encoding the oxalate:formate antiporter [83]. All of these genomes were isolated from *A. mellifera*, suggesting a potential role in mediating symbiosis with this particular host species. One possibility is that oxalate uptake by *Gilliamella* prevents oxalate from harming its host, as topical application of oxalate to the *A. mellifera* had been evidenced to result in cell apoptosis [84]. However, no genes for oxalate metabolism could be identified from any of the 95 genomes. Therefore, it is yet known how *Gilliamella* may further metabolize this substrate. Formate may also be metabolized by *Gilliamella*, as genes encoding three enzymes – an anaerobic ribonucleoside-triphosphate reductase, 7,8-dihydroneopterin oxygenase and formate-tetrahydrofolate ligase – could also be found in all 95 *Gilliamella* genomes, all of which catalyse reactions with formate as a substrate. This also suggests that a lack of formate detection alongside lactate production [11] could also occur even if formate was produced as a byproduct of fermentation.

There is ample evidence to suggest that *Gilliamella* can utilize succinate for a variety of anabolic pathways. The genes encoding the succinyl-CoA synthetase complex that catalyses the conversion of succinate to succinyl-CoA [85] were found in all 95 genomes, as do genes necessary for further conversion to l-lysine or l-methionine via their respective biosynthesis pathways [86,87]. Genes were also found to suggest *Gilliamella* can utilize succinate alongside l-cystathionine to synthesize l-cysteine, with all genes necessary for synthesizing l-cystathionine from oxaloacetate via l-aspartate and l-homoserine found in all 95 genomes examined. As consistent with previous research of *G. apicola* [15] and as aforementioned, genes encoding the TCA cycle are severely lacking or almost completely absent in the 95 *Gilliamella* genomes examined. Therefore, these pathways may represent alternative methods of *de novo* amino acid biosynthesis. However, the entanglement of such biosynthesis pathways with microaerophilic respiration may mean that biosynthesis of these amino acids is highly dependent on changing oxic conditions within the host gut environment.

It is unclear what happens to the protoporphyrin IX product of microaerophilic respiration, given the lack of genes found for further conversions as previously mentioned. Amides derived from protoporphyrin have been found to play antagonistic roles against bee pathogens [88]. Co-metabolism and biosynthesis have been described for *Gilliamella* and *Snodgrassella* [15], so it may be of interest to determine if protoporphyrin IX potentially produced by *Gilliamella* may be further utilized for the biosynthesis of bioactive compounds.

The potential capacity and preference to utilize the (*R*)-lactate, acetate, formate and succinate products of energy metabolism, along with regeneration of NAD(P)H, suggests that *Gilliamella* may be adept at conserving resources and may also explain why a lack of acetoin and ethanol synthesized by *Gilliamella* was characterized [11]. Interestingly, *Gilliamella* is also known to specifically provision formate, lactate, pyruvate and succinate to the co-habiting *Snodgrassella* [89] (Fig. 2). This may further explain the preference for fermentation pathways resulting in these substrates, though in such cases, the regulatory mechanism is currently unknown. Maximizing resource utilization may confer fitness advantages, allowing for better scavenging of energy sources and, therefore, better competition against other micro-organisms that can engage in similar metabolic processes, including lactic acid bacteria [11,90].

### The energy metabolism pathways of *Gilliamella* are also conserved within the *Orbaceae*

*Gilliamella* energy metabolism had previously been studied at different scopes and scales, both *in silico* and *in vitro*. However, to date, this is the first study that reconstructed the genomic features underlying energy metabolism pathways across a significant number of genomes representing diverse *Gilliamella* species from diverse hosts. Here, genetic evidence suggests that the core energy metabolism processes employed are highly conserved, regardless of the *Gilliamella* species/strain or host taxa, as consistent with what is suggested by the recent pan-genomic study [91]. Specifically, genetic evidence suggests that the glycolysis and pentose phosphate pathways are the predominant mechanisms through which *Gilliamella* processes sugars and derivatives as energy sources, feeding into consistent fermentation and respiratory pathways, with the latter occurring solely under microoxic conditions, and that the TCA cycle likely only plays an incidental role in energy metabolism, if at all. Products of energy metabolism are also generally consistent, as are a number of potential energy sources – namely glucose, fructose, arbutin, salicin, cellobiose and glucosamine.

Previous studies had found evidence that some of the components of *Gilliamella* energy metabolism are also shared with other *Orbaceae*, with ubiquinone-8 biosynthesis shared with all other members of this family [32,35], and specifically the lack of genes involved in the TCA cycle and the lack of *nuo* genes with *Ca*. S. bombi Bimp [32]. Further examinations in this study found that beyond the two copies of *ptsH*, the genomic features underpinning other energy metabolism processes were also indeed abundantly present in all other *Orbaceae* genomes. Genes for fermentation pathways from pyruvate to (*R*)-lactate, ethanol and acetate were found in all *Orbaceae* genomes, including with formate as a potential byproduct, and to (*S*)-lactate in *Z. entericus* and *F. perrara* genomes, suggesting that fermentation pathways were also largely conserved within the *Orbaceae*. Interestingly, genes necessary for the fermentation of pyruvate to acetoin were also found in all other *Orbaceae* genomes. This suggests that this capacity had arisen early in the evolution of the family and then lost in the majority of *Gilliamella*. The specific enzymes/enzymatic complexes involved in microaerophilic, but not fully aerobic respiration, were also highly consistent across *Orbaceae* species, including the lack of *ubiC* in all genomes examined, suggesting again that these features are conserved across the family, although *narGHI* were also found in the genomes of *Ca*. S. bombi, *O. hercynius* and *Z. entericus*. Genes encoding the partial TCA cycle catalysing the conversion of acetyl-CoA and oxaloacetate to 2-oxoglutarate could also be found for *O. hercynius* and *Z. entericus*, but not for *Ca*. S. bombi or *F. perrara*.

The genes that are necessary for the uptake and metabolism of glucose, fructose, arbutin, salicin, cellobiose and glucosamine via the PTS found in all *Gilliamella* genomes were also found in all other *Orbaceae* genomes, except for *nagE* in *O. hercynius*. These sugars and derivatives are known to be present in or are derived from plant matter consumed by bees [92]; thus, the capacity to metabolize these substrates may be particularly beneficial to bee symbionts and may explain why multiple *Orbaceae* genera have formed symbiotic relationships with bee hosts.

Although almost all of the *Gilliamella* strains in this study were only isolated from the bee hindgut, the consistency of genomic features characterized not only across the genus but also for other *Orbaceae* suggests that the energy metabolism pathways characterized in this study are truly representative of the genus and not reflective of strains specifically adapted to the host gut environment. It is likely then that the core energy metabolism features conserved across *Gilliamella* acted, and continue to act, as pre-adaptations to symbiosis with corbiculate bee hosts in general, while capabilities for uptake and metabolism of specific sugars and derivatives may possibly represent adaptations to specific host taxa. This could also explain the apparent contradictions in findings of host-specificity of *Gilliamella* [63,93,95], with the pre-adaptations allowing multiple strains or even species of *Gilliamella* to colonize the guts of corbiculate bees post-disturbance, but then selection pressure reduces the diversity of *Gilliamella* to just the species/strain best adapted to symbiosis with the specific host bee species. This could also explain why *Gilliamella* did not seem to have entirely co-evolved with corbiculate bee hosts [96] if symbiotic relationships could be readily formed and another species of *Gilliamella* could entirely replace an existing one. The flexibility in energy metabolism capabilities may also explain why *Gilliamella* seems to respond better to changes in the host environment, including due to changes caused by host ageing, compared with other symbionts [62,97]. The specific differential patterns of energy metabolism across *Orbaceae* are also of interest, as the distribution of the presence/absence of certain traits does not seem to line up entirely with known *Orbaceae* phylogeny. However, this is out of the scope of this study and could be explored in a future study.

## Conclusion

Energy metabolism can play an important role in dictating the lifestyles of an organism, especially in the context of oxic conditions required for respiration. This has driven significant interest in the energy metabolism patterns of *Gilliamella*, finding much diversity across different *Gilliamella* species and strains. Here, 95 *Gilliamella* genomes were examined to determine the underlying pathways for energy metabolism and the differences and similarities across the entire genus, expanding on existing research. It was found that energy metabolism capacities were largely identical across the entire genus, with all species likely capable of almost identical fermentation and microaerophilic respiration activities, along with comprising identical capabilities for uptake and metabolism of a select number of sugars and derivatives. The main differences were in additional types of sugars and derivatives that may reflect adaptations in some cases to symbiosis with specific corbiculate bee taxa, along with the capacity for fermentation to acetoin for some *G. apicola*.

There are some limitations that should be considered, however. This study focused on genomic features involved in energy metabolism, and while this study found such features were certainly conserved across the genus, further research is required to determine if it is the same with other characteristics of *Gilliamella* biology. It is also important for features described to be confirmed *in vivo* or *in vitro*. Additionally, not all publicly available *Gilliamella* genomes were examined in this study, and likely, there are more *Gilliamella* species that have yet to be sequenced. Further investigations are also necessary to determine what differences in features between *Gilliamella* and other *Orbaceae* beyond those already described were retained or lost during the evolution of the various taxa within the family.

Nonetheless, the knowledge of *Gilliamella* energy metabolisms that could be described in this study has important implications for future research, especially in the context of *Gilliamella* activities outside the host environment in fully oxic conditions, but also in relation to potential capacities for sugar metabolism and how such processes may shape *Gilliamella* ecological roles and evolution. The conserved nature of genes involved in respiration, despite evidence of genomic reduction and loss of function, suggests they may nonetheless play an important yet undescribed role and could be of further research interest. The evolution of *Gilliamella*, especially in the context of *Orbaceae* as a whole, could also be of interest, given that evidence in this study suggests the various *Orbaceae* taxa may be significantly more closely related than previously suggested.

## supplementary material

10.1099/acmi.0.000793.v3 Uncited Supplementary Material 1.

## Abbreviations

ATPase: ATP synthase
COG: Clusters of Orthologous Group
CytBD: cytochrome-bd oxidase complex
ETC: electron transport chain
GTDB: Genome Taxonomy Database
HGT: horizontal genetic transfer
IMG/M: integrated microbial genomes and microbiomes
KEGG: Kyoto Encyclopedia of Genes and Genomes
NADH: nicotinamide adenine dinucleotide
NADPH: nicotinamide adenine dinucleotide phosphate
NCBI: National Center for Biotechnology Information
PTS: phosphoenol-pyruvate-dependent phosphotransferase system
TCA cycle: tricarboxylic acid cycle
