# Supplementary material for: Genomic investigations of diverse corbiculate bee gut-associated Gilliamella reveal conserved pathways for energy metabolism, with diverse and variable energy sources
Source: Access Microbiol. 2024 Aug 15;6(8):000793.v3. doi: 10.1099/acmi.0.000793.v3 (PMC11325843; doi:10.1099/acmi.0.000793.v3)
Supplement: Uncited Supplementary Material 1. [file acmi-6-00793-s001.pdf]

Supplementary Tables

Supplementary Table S1: Non-*Gilliamella* genomes from the family *Orbaceae* in the IMG/M database, as of April 24th 2023.

**IMG genome ID**	**Strain**
2505679062	*Candidatus* Schmidhempelia bombi Bimp
2585427711	*Candidatus* Schmidhempelia bombi Bimp
2756170266	*Frischella perrara* DSM 104328
2833532623	*Frischella perrara* ESL0167
2684622921	*Frischella perrara* Fp_167
2630968947	*Frischella perrara* PEB0191
2515154034	*Frischella perrara* PEB0191
2770939645	*Orbus hercynius* DSM 22228
2883515056	*Zophobihabitans entericus* IPMB12

Supplementary Table S2: Metadata for each genome in this study, including the NCBI accession number, NCBI taxonomy (species), and GTDB taxonomy (species), host, and isolation source. Four genomes had their host and isolation sources identified from literature as cited next to the NCBI accession number.

NCBI accession	NCBI taxonomy	GTDB taxonomy	Host	Isolation source
GCF_002141665.1	*Gilliamella apicola*	*Gilliamella apicola_N*	*Apis mellifera*	gut
GCF_002141655.1	*Gilliamella apicola*	*Gilliamella apicola*	*Apis mellifera*	gut
GCF_002141565.1	*Gilliamella apicola*	*Gilliamella apicola*	*Apis mellifera*	gut
GCF_002141675.1	*Gilliamella apicola*	*Gilliamella apicola_N*	*Apis mellifera*	gut
GCF_002141515.1	*Gilliamella apicola*	*Gilliamella apicola*	*Apis mellifera*	gut
GCF_002141575.1	*Gilliamella apicola*	*Gilliamella apicola_N*	*Apis mellifera*	gut
GCF_002141905.1	*Gilliamella apicola*	*Gilliamella apicola_N*	*Apis mellifera*	gut
GCF_002141555.1	*Gilliamella apicola*	*Gilliamella apicola*	*Apis mellifera*	gut
GCF_002141745.1	*Gilliamella apicola*	*Gilliamella apicola*	*Apis mellifera*	gut
GCF_002141715.1	*Gilliamella apicola*	*Gilliamella apicola_N*	*Apis mellifera*	gut
GCF_002141815.1	*Gilliamella apicola*	*Gilliamella apicola_N*	*Apis mellifera*	gut
GCF_002141595.1	*Gilliamella apicola*	*Gilliamella apicola_N*	*Apis mellifera*	gut
GCF_001690495.1	*Gilliamella apicola*	*Gilliamella apicola_G*	*Bombus appositus*	gut
GCF_001690255.1	*Gilliamella apicola*	*Gilliamella apicola_G*	*Bombus appositus*	gut
GCF_001690355.1	*Gilliamella apicola*	*Gilliamella apicola_A*	*Bombus impatiens*	gut
GCF_001690585.1	*Gilliamella apicola*	*Gilliamella apicola_A*	*Bombus impatiens*	gut
GCF_001690835.1	*Gilliamella apicola*	*Gilliamella bombi*	*Bombus bimaculatus*	gut
GCF_003202815.1	*Gilliamella apicola*	*Gilliamella apicola*	*Apis mellifera*	gut
GCF_003202915.1	*Gilliamella apicola*	*Gilliamella apicola_Q*	*Apis mellifera*	gut
GCF_001690385.1	*Gilliamella apicola*	*Gilliamella apicola_A*	*Bombus impatiens*	gut
GCF_001690595.1	*Gilliamella apicola*	*Gilliamella apicola_M*	*Bombus impatiens*	gut
GCF_001690515.1	*Gilliamella apicola*	*Gilliamella apicola_C*	*Bombus fervidus*	gut
GCF_001690825.1	*Gilliamella apicola*	*Gilliamella apicola_C*	*Bombus fervidus*	gut
GCF_003202655.1	*Gilliamella apicola*	*Gilliamella apicola_Q*	*Apis mellifera*	gut
GCF_001690335.1	*Gilliamella apicola*	*Gilliamella bombi*	*Bombus griseocollis*	gut

GCF_001690815.1	*Gilliamella apicola*	*Gilliamella bombi*	*Bombus impatiens*	gut
GCF_001690425.1	*Gilliamella apicola*	*Gilliamella bombi*	*Bombus impatiens*	gut
GCF_001690795.1	*Gilliamella apicola*	*Gilliamella apicola_C*	*Bombus pensylvanicus*	gut
GCF_001690265.1	*Gilliamella apicola*	*Gilliamella apicola_C*	*Bombus pensylvanicus*	gut
GCF_001690345.1	*Gilliamella apicola*	*Gilliamella bombi*	*Bombus griseocollis*	gut
GCF_002088895.1	*Gilliamella apicola*	*Gilliamella apicola*	*Apis mellifera*	gut
GCF_002142345.1	*Gilliamella apicola*	*Gilliamella apicola*	*Apis mellifera*	gut
GCF_002142285.1	*Gilliamella apicola*	*Gilliamella apicola*	*Apis mellifera*	gut
GCF_002088905.1	*Gilliamella apicola*	*Gilliamella apicola*	*Apis mellifera*	gut
GCF_002088825.1	*Gilliamella apicola*	*Gilliamella apicola*	*Apis mellifera*	gut
GCF_001690435.1	*Gilliamella apicola*	*Gilliamella apicola_C*	*Bombus nevadensis*	gut
GCF_002141885.1	*Gilliamella apicola*	*Gilliamella apicola_N*	*Apis mellifera*	gut
GCF_002141855.1	*Gilliamella apicola*	*Gilliamella apicola_N*	*Apis mellifera*	gut
GCF_002141785.1	*Gilliamella apicola*	*Gilliamella apicola_N*	*Apis mellifera*	gut
GCF_002142265.1	*Gilliamella apicola*	*Gilliamella apicola*	*Apis mellifera*	gut
GCF_002088795.1	*Gilliamella apicola*	*Gilliamella apicola*	*Apis mellifera*	gut
GCF_001690895.1	*Gilliamella apicola*	*Gilliamella apicola_C*	*Bombus nevadensis*	gut
GCF_001690735.1	*Gilliamella apicola*	*Gilliamella apicola*	*Apis mellifera*	gut
GCF_001690675.1	*Gilliamella apicola*	*Gilliamella bombi*	*Bombus occidentalis*	gut
GCF_001690655.1	*Gilliamella apicola*	*Gilliamella apicola_P*	*Bombus occidentalis*	gut
GCF_002088815.1	*Gilliamella apicola*	*Gilliamella apicola*	*Apis mellifera*	gut
GCF_000733115.1	*Gilliamella apicola*	*Gilliamella apicola_A*	*Bombus bimaculatus*	gut
GCF_001690195.1	*Gilliamella apicola*	*Gilliamella apicola_L*	*Apis dorsata*	gut
GCF_000599985.1	*Gilliamella apicola*	*Gilliamella apicola*	*Apis mellifera*	gut
GCF_001690275.1	*Gilliamella apicola*	*Gilliamella apicola_C*	*Bombus pensylvanicus*	gut
GCF_001690445.1	*Gilliamella apicola*	*Gilliamella apicola_E*	*Apis cerana*	gut
GCF_001690875.1	*Gilliamella apicola*	*Gilliamella apicola_A*	*Bombus vagans*	gut

GCF_001690705.1	*Gilliamella apicola*	*Gilliamella apicola_K*	*Apis dorsata*	gut
GCF_001693755.1	*Gilliamella apicola*	*Gilliamella apicola_F*	*Apis andreniformis*	gut
GCF_001693745.1	*Gilliamella apicola*	*Gilliamella apicola_E*	*Apis cerana*	gut
GCF_001693435.1	*Gilliamella apicola*	*Gilliamella apicola_N*	*Apis mellifera*	gut
GCF_001690185.1	*Gilliamella apicola*	*Gilliamella apicola_I*	*Apis cerana*	gut
GCF_000695585.1	*Gilliamella apicola*	*Gilliamella bombi*	*Bombus vagans*	gut
GCF_002142135.1	*Gilliamella apis*	*Gilliamella apis*	*Apis mellifera*	gut
GCF_002142065.1	*Gilliamella apis*	*Gilliamella apis*	*Apis mellifera*	gut
GCF_002142055.1	*Gilliamella apis*	*Gilliamella apis*	*Apis mellifera*	gut
GCF_002142035.1	*Gilliamella apis*	*Gilliamella apis*	*Apis mellifera*	gut
GCF_002141865.1	*Gilliamella apis*	*Gilliamella apis*	*Apis mellifera*	gut
GCF_002142185.1	*Gilliamella apis*	*Gilliamella apis*	*Apis mellifera*	gut
GCF_001690775.1	*Gilliamella apis*	*Gilliamella apis*	*Apis mellifera*	gut
GCF_003202775.1	*Gilliamella apis*	*Gilliamella apis_A*	*Apis mellifera*	gut
GCF_003202875.1	*Gilliamella apis*	*Gilliamella apis*	*Apis mellifera*	gut
GCF_002141825.1	*Gilliamella apis*	*Gilliamella apis*	*Apis mellifera*	gut
GCF_002142085.1	*Gilliamella apis*	*Gilliamella apis*	*Apis mellifera*	gut
GCF_002141945.1	*Gilliamella apis*	*Gilliamella apis*	*Apis mellifera*	gut
GCF_002142255.1	*Gilliamella apis*	*Gilliamella apis*	*Apis mellifera*	gut
GCF_002142225.1	*Gilliamella apis*	*Gilliamella apis*	*Apis mellifera*	gut
GCF_002142165.1	*Gilliamella apis*	*Gilliamella apis*	*Apis mellifera*	gut
GCF_002142155.1	*Gilliamella apis*	*Gilliamella apis*	*Apis mellifera*	gut
GCF_002141985.1	*Gilliamella apis*	*Gilliamella apis*	*Apis mellifera*	gut
GCF_002141935.1	*Gilliamella apis*	*Gilliamella apis*	*Apis mellifera*	gut
GCF_002142115.1	*Gilliamella apis*	*Gilliamella apis*	*Apis mellifera*	gut
GCF_900094935.1 [1]	*Gilliamella intestini*	*Gilliamella intestini*	*Bombus lapidarius*	gut + crop
GCF_900094945.1 [1]	*Gilliamella bombicola*	*Gilliamella bombicola*	*Bombus pascuorum + Bombus lapidarius*	gut + crop
GCF_900103255.1	*Gilliamella bombi*	*Gilliamella bombi*	*Bombus terrestris*	gut + crop

[1]				
GCF_001690175.1	*Gilliamella apis*	*Gilliamella apis*	*Apis mellifera*	gut
GCF_001690755.1	*Gilliamella apis*	*Gilliamella apis*	*Apis mellifera*	gut
GCF_900103085.1 [1]	*Gilliamella mensalis*	*Gilliamella mensalis*	*Bombus pascuorum*	gut + crop
GCF_002142275.1	*Gilliamella sp. N-W3*	*Gilliamella sp002142215*	*Apis mellifera*	gut
GCF_002142215.1	*Gilliamella sp. N-G2*	*Gilliamella sp002142215*	*Apis mellifera*	gut
GCF_009795905.1	*Gilliamella sp. Lep-s5*	*Gilliamella sp009795845*	*Bombus lepidus*	gut
GCF_009795845.1	*Gilliamella sp. Lep-s35*	*Gilliamella sp009795845*	*Bombus lepidus*	gut
GCF_009795895.1	*Gilliamella sp. Lep-s21*	*Gilliamella sp009795845*	*Bombus lepidus*	gut
GCF_009795745.1	*Gilliamella sp. Pra-s65*	*Gilliamella sp009795805*	*Bombus pratorum*	gut
GCF_009795755.1	*Gilliamella sp. Pra-s60*	*Gilliamella sp009795805*	*Bombus pratorum*	gut
GCF_009795785.1	*Gilliamella sp. Pra-s54*	*Gilliamella sp009795805*	*Bombus pratorum*	gut
GCF_009795805.1	*Gilliamella sp. Pra-s52*	*Gilliamella sp009795805*	*Bombus pratorum*	gut
GCF_009795795.1	*Gilliamella sp. Pas-s95*	*Gilliamella bombicola*	*Bombus pascuorum*	gut
GCF_009795865.1	*Gilliamella sp. Pas-s27*	*Gilliamella sp009795805*	*Bombus pascuorum*	gut
GCF_009795885.1	*Gilliamella sp. Pas-s25*	*Gilliamella sp009795885*	*Bombus pascuorum*	gut

Supplementary Figures

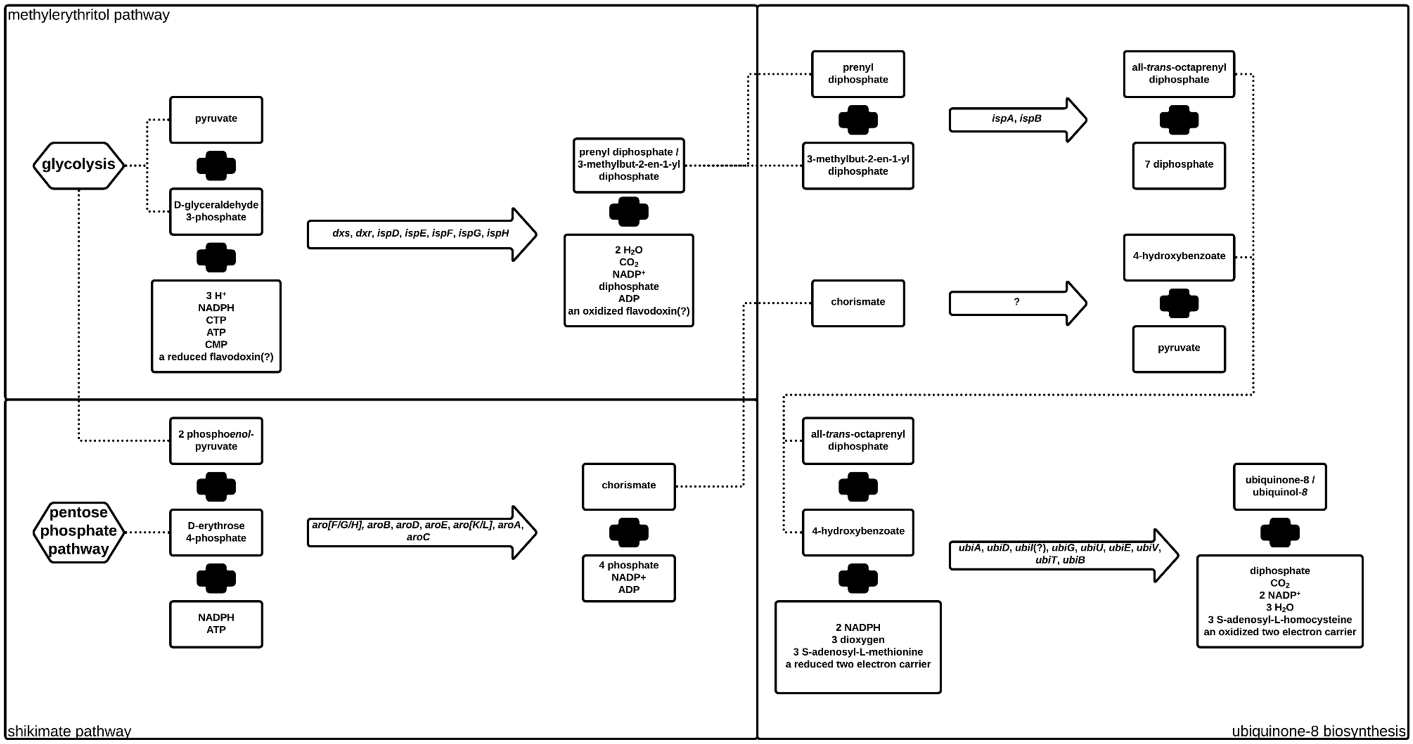

Supplementary Figure S1: Proposed ubiquinone/ubiquinol-8 biosynthesis pathway employed by bee gut-associated *Gilliamella* strains. All genes involved are listed. Ambiguous gene names are listed in square brackets. ‘?’ by itself means the gene(s) involved could not be identified. ‘?’ in brackets indicates its involvement is unclear.

(A)

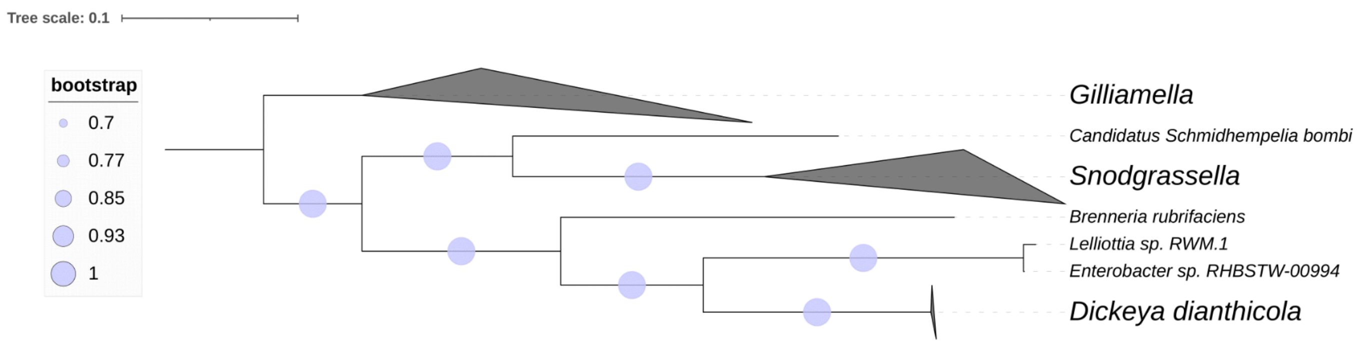
Tree scale: 0.1
bootstrap
0.7
0.77
0.85
0.93
1
Gilliamella
Candidatus Schmidhempelia bombi
Snodgrassella
Brenneria rubrifaciens
Lelliottia sp. RWM.1
Enterobacter sp. RHBSTW-00994
Dickeya dianthicola

(B)

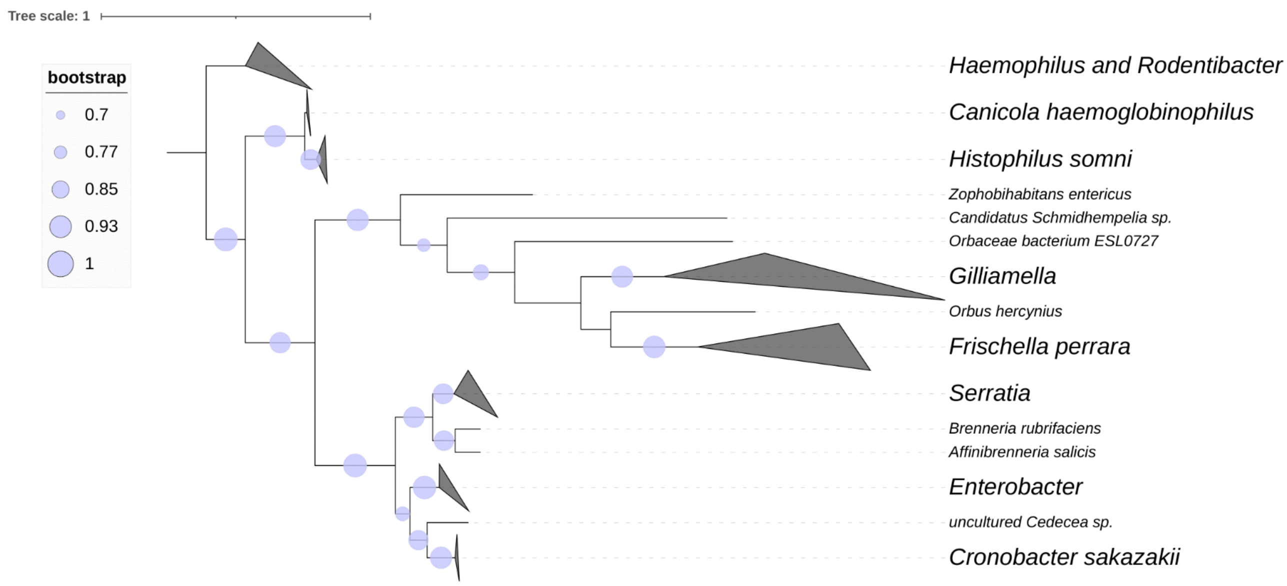
Tree scale: 1
bootstrap
0.7
0.77
0.85
0.93
1
Haemophilus and Rodentibacter
Canicola haemoglobinophilus
Histophilus somni
Zophobihabitans entericus
Candidatus Schmidhempelia sp.
Orbaceae bacterium ESL0727
Gilliamella
Orbus hercynius
Frischella perrara
Serratia
Brenneria rubrifaciens
Affinibrenneria salicis
Enterobacter
uncultured Cedecea sp.
Cronobacter sakazakii

(C)

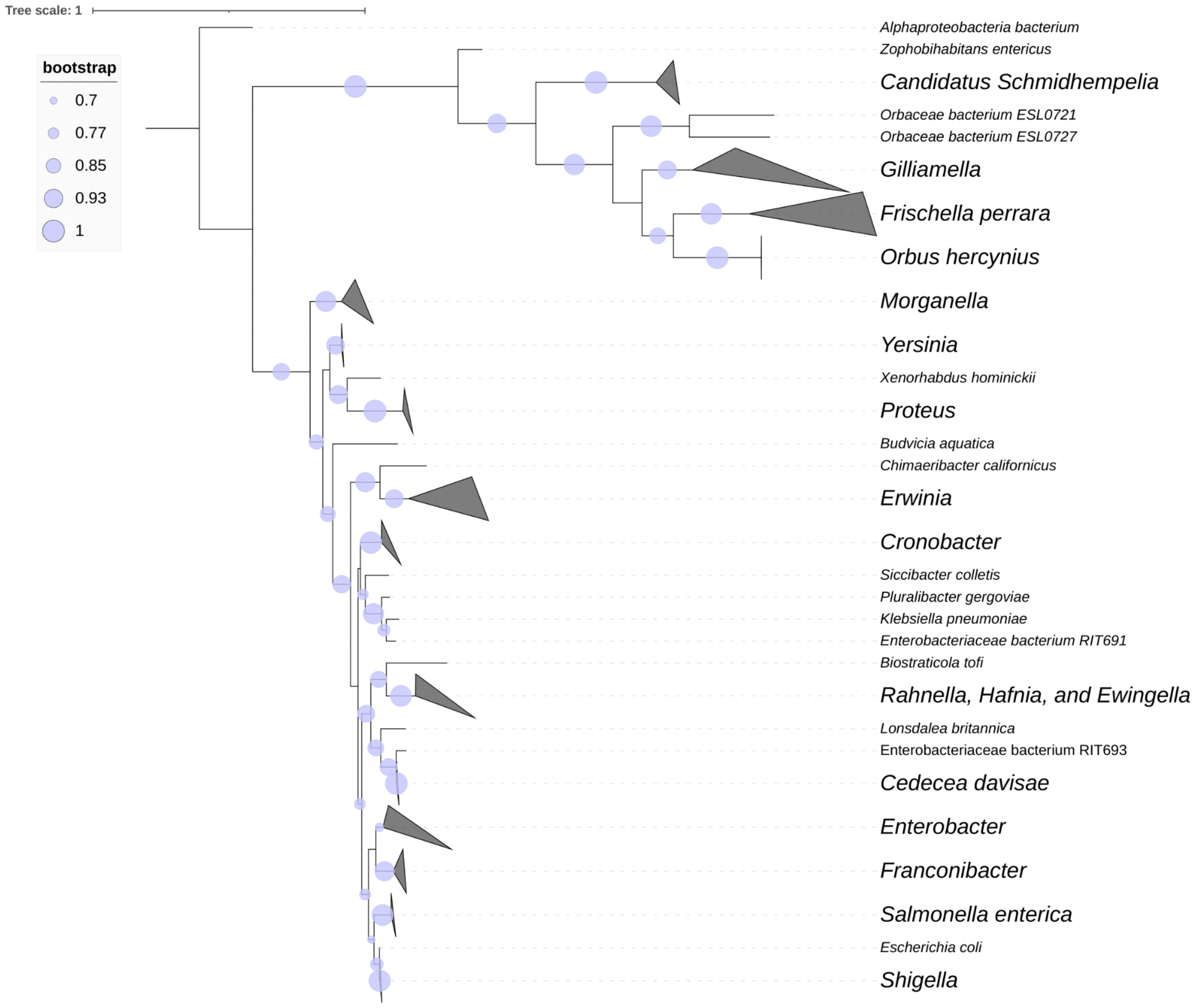

Supplementary Figure S2: Phylogenetic analysis of the *frd*, *frdC*, and *frdD* genes found in the 95 *Gilliamella* genomes in this study, along with those in other *Orbaceae* genomes in the IMG/M database, and top 250 BLASTp hits from the NCBI database (as of August 27th, 2023). Formatting and annotation was done via iTOL.

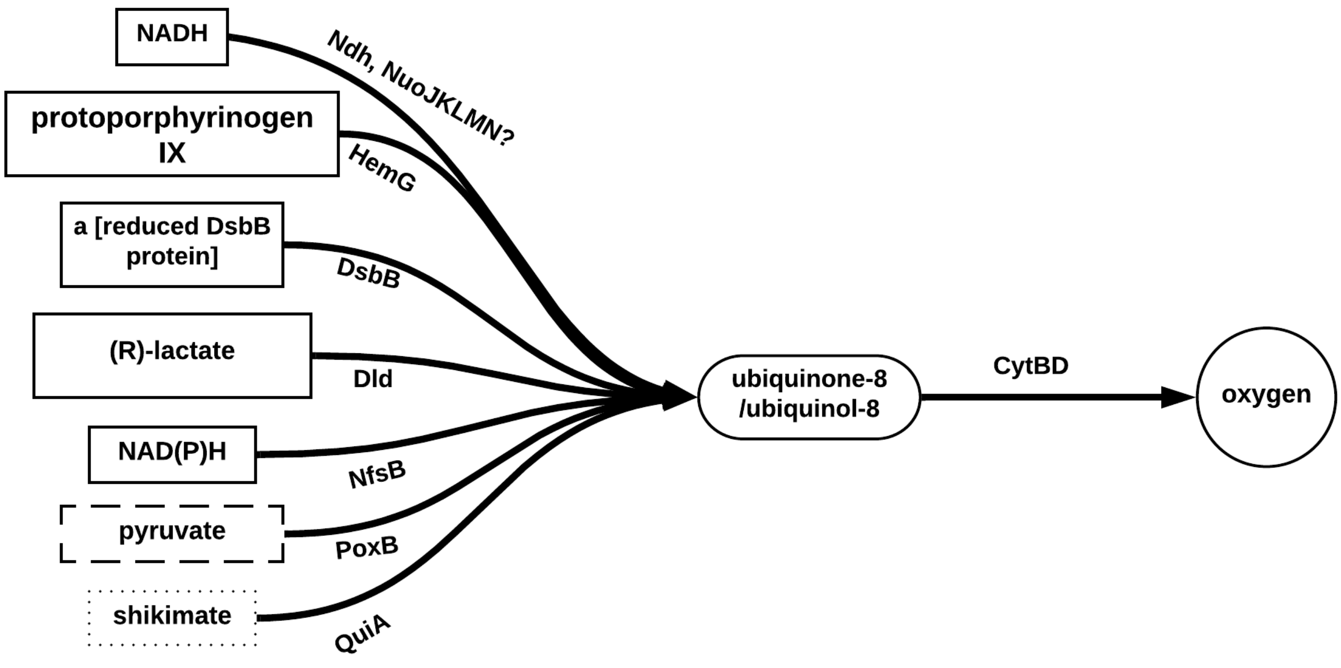

Supplementary Figure S3: Proposed *Gilliamella* electron transport chain based on genes identified. Electron donors are represented with rectangles, electron-transfer quinone in an oval, and electron acceptors with circles. Arrows indicate directional flow of electrons, accompanied with the name of the enzyme (complex) that catalyzes the reaction. Rectangles with disjointed and dotted edges respectively indicate the gene allowing for utilization of the respective electron donor can be found in 87 and 7 genomes.

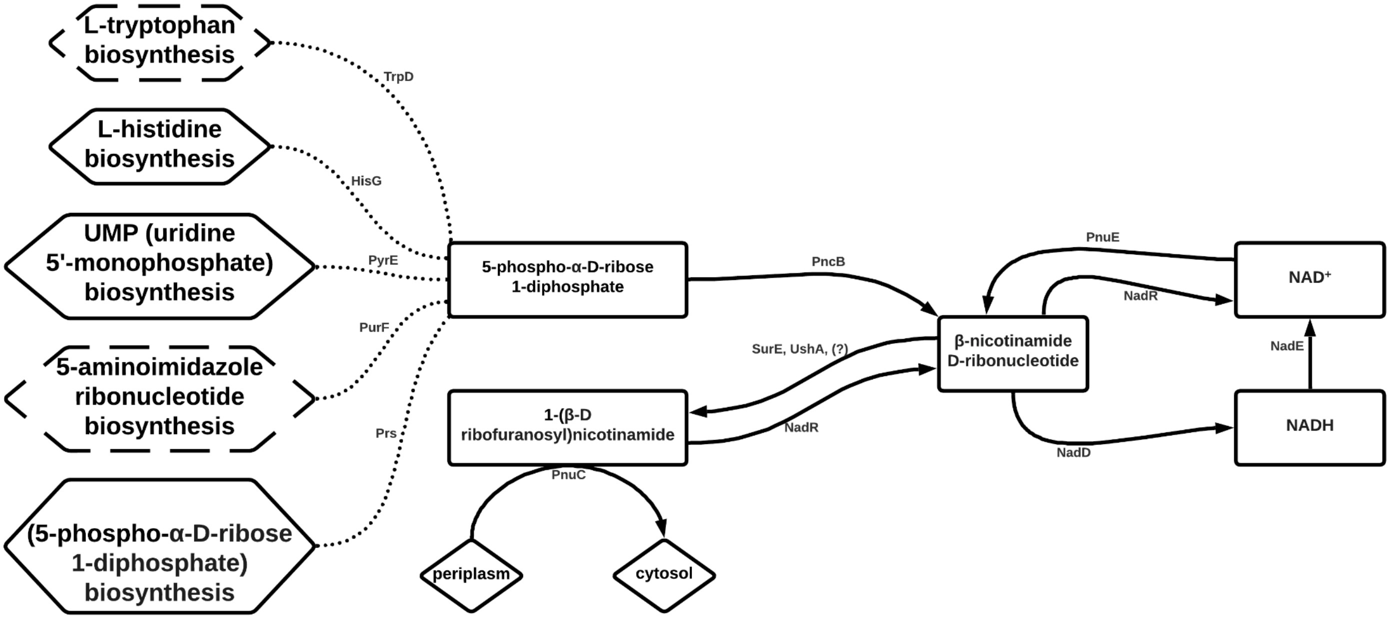

Supplementary Figure S4: Pathway for biosynthesis of NAD(H) by *Gilliamella* based on genes identified. Genes encoding the enzymes were found in all genomes, except for *ushA*, *surE*, and one encoding a 5'-nucleotidase without a gene name (K01081) that were found in 94, 70, and 7 genomes respectively. Pathways of 5-phospho-α-D-ribose 1-diphosphate biosynthesis either has a solid edge, indicating the production of this molecule is unidirectional, or has a broken edge, indicating the enzymatic reaction is bidirectional.

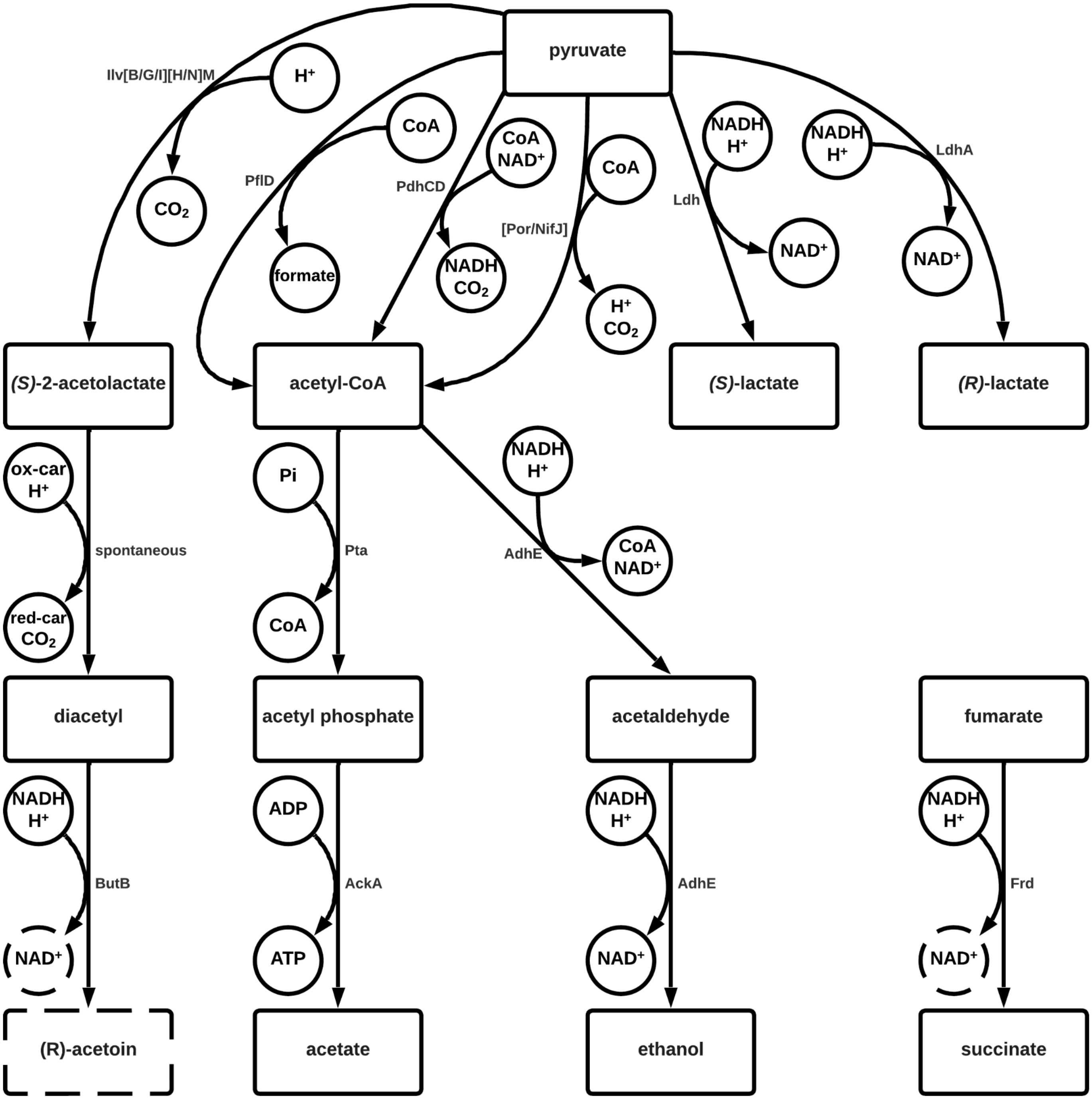

Supplementary Figure S5: Proposed fermentation pathways by *Gilliamella*. Genes encoding all enzymes listed were identified from all 95 *Gilliamella* genomes, except for ButB which was only from 16 genomes, reflected by complete and broken lines for the products respectively. CoA = coenzyme A, ox-car = oxidized carrier, red-car = reduced carrier, Pi = phosphate.

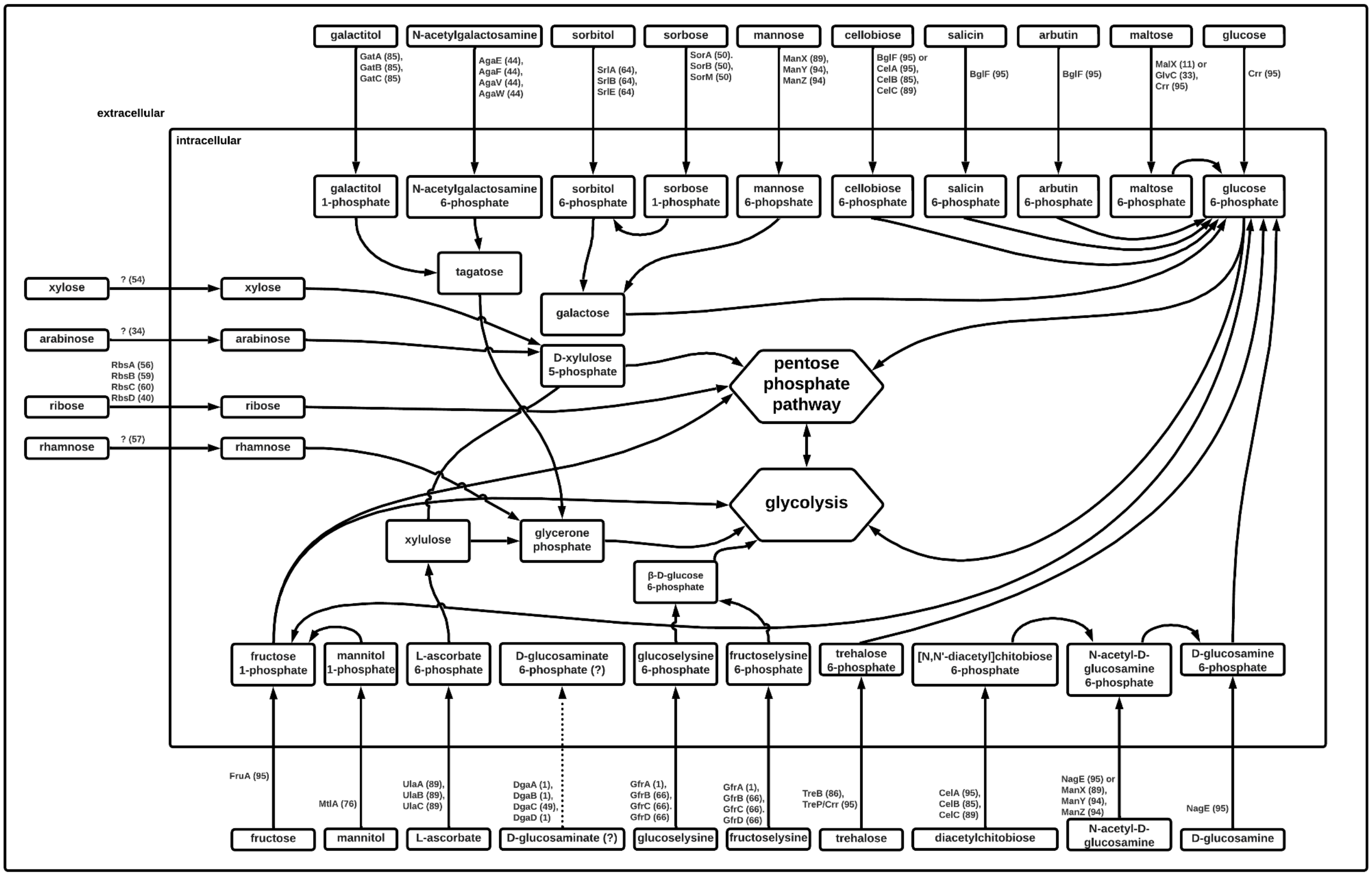

Supplementary Figure S6: Proposed pathways for uptake and metabolism of diverse sugars and derivatives via the glycolysis and pentose phosphate pathways. Ribose transport is via an ABC transporter and requires only the genes listed. In all other cases, transport is via the phosphoenol-pyruvate-dependent phosphotransferase system and requires *ptsHI* in addition to the genes listed. Numbers in brackets is the number of genomes the gene could be identified from. The dotted line for D-glucosaminate import indicates the capacity for *Gilliamella* to uptake this substrate to be unlikely despite genetic evidence identified. A '?' indicates the gene(s) responsible for transport of the respective substrate is unknown, despite the likelihood of it occurring.

(A)

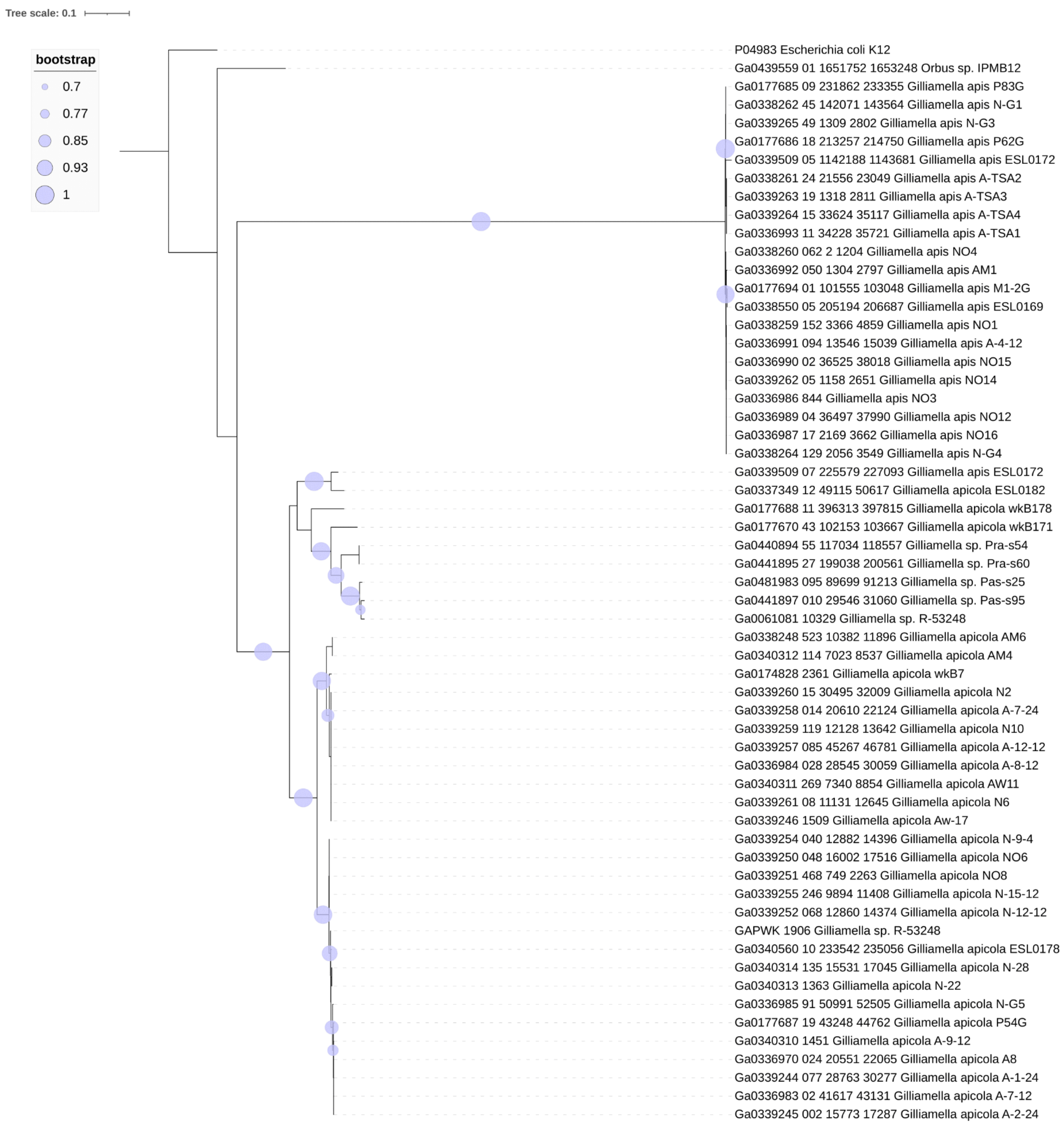
Tree scale: 0.1
bootstrap
0.7
0.77
0.85
0.93
1
P04983 Escherichia coli K12
Ga0439559 01 1651752 1653248 Orbus sp. IPMB12
Ga0177685 09 231862 233355 Gilliamella apis P83G
Ga0338262 45 142071 143564 Gilliamella apis N-G1
Ga0339265 49 1309 2802 Gilliamella apis N-G3
Ga0177686 18 213257 214750 Gilliamella apis P62G
Ga0339509 05 1142188 1143681 Gilliamella apis ESL0172
Ga0338261 24 21556 23049 Gilliamella apis A-TSA2
Ga0339263 19 1318 2811 Gilliamella apis A-TSA3
Ga0339264 15 33624 35117 Gilliamella apis A-TSA4
Ga0336993 11 34228 35721 Gilliamella apis A-TSA1
Ga0338260 062 2 1204 Gilliamella apis NO4
Ga0336992 050 1304 2797 Gilliamella apis AM1
Ga0177694 01 101555 103048 Gilliamella apis M1-2G
Ga0338550 05 205194 206687 Gilliamella apis ESL0169
Ga0338259 152 3366 4859 Gilliamella apis NO1
Ga0336991 094 13546 15039 Gilliamella apis A-4-12
Ga0336990 02 36525 38018 Gilliamella apis NO15
Ga0339262 05 1158 2651 Gilliamella apis NO14
Ga0336986 844 Gilliamella apis NO3
Ga0336989 04 36497 37990 Gilliamella apis NO12
Ga0336987 17 2169 3662 Gilliamella apis NO16
Ga0338264 129 2056 3549 Gilliamella apis N-G4
Ga0339509 07 225579 227093 Gilliamella apis ESL0172
Ga0337349 12 49115 50617 Gilliamella apicola ESL0182
Ga0177688 11 396313 397815 Gilliamella apicola wkB178
Ga0177670 43 102153 103667 Gilliamella apicola wkB171
Ga0440894 55 117034 118557 Gilliamella sp. Pra-s54
Ga0441895 27 199038 200561 Gilliamella sp. Pra-s60
Ga0481983 095 89699 91213 Gilliamella sp. Pas-s25
Ga0441897 010 29546 31060 Gilliamella sp. Pas-s95
Ga0061081 10329 Gilliamella sp. R-53248
Ga0338248 523 10382 11896 Gilliamella apicola AM6
Ga0340312 114 7023 8537 Gilliamella apicola AM4
Ga0174828 2361 Gilliamella apicola wkB7
Ga0339260 15 30495 32009 Gilliamella apicola N2
Ga0339258 014 20610 22124 Gilliamella apicola A-7-24
Ga0339259 119 12128 13642 Gilliamella apicola N10
Ga0339257 085 45267 46781 Gilliamella apicola A-12-12
Ga0336984 028 28545 30059 Gilliamella apicola A-8-12
Ga0340311 269 7340 8854 Gilliamella apicola AW11
Ga0339261 08 11131 12645 Gilliamella apicola N6
Ga0339246 1509 Gilliamella apicola Aw-17
Ga0339254 040 12882 14396 Gilliamella apicola N-9-4
Ga0339250 048 16002 17516 Gilliamella apicola NO6
Ga0339251 468 749 2263 Gilliamella apicola NO8
Ga0339255 246 9894 11408 Gilliamella apicola N-15-12
Ga0339252 068 12860 14374 Gilliamella apicola N-12-12
GAPWK 1906 Gilliamella sp. R-53248
Ga0340560 10 233542 235056 Gilliamella apicola ESL0178
Ga0340314 135 15531 17045 Gilliamella apicola N-28
Ga0340313 1363 Gilliamella apicola N-22
Ga0336985 91 50991 52505 Gilliamella apicola N-G5
Ga0177687 19 43248 44762 Gilliamella apicola P54G
Ga0340310 1451 Gilliamella apicola A-9-12
Ga0336970 024 20551 22065 Gilliamella apicola A8
Ga0339244 077 28763 30277 Gilliamella apicola A-1-24
Ga0336983 02 41617 43131 Gilliamella apicola A-7-12
Ga0339245 002 15773 17287 Gilliamella apicola A-2-24

(B)

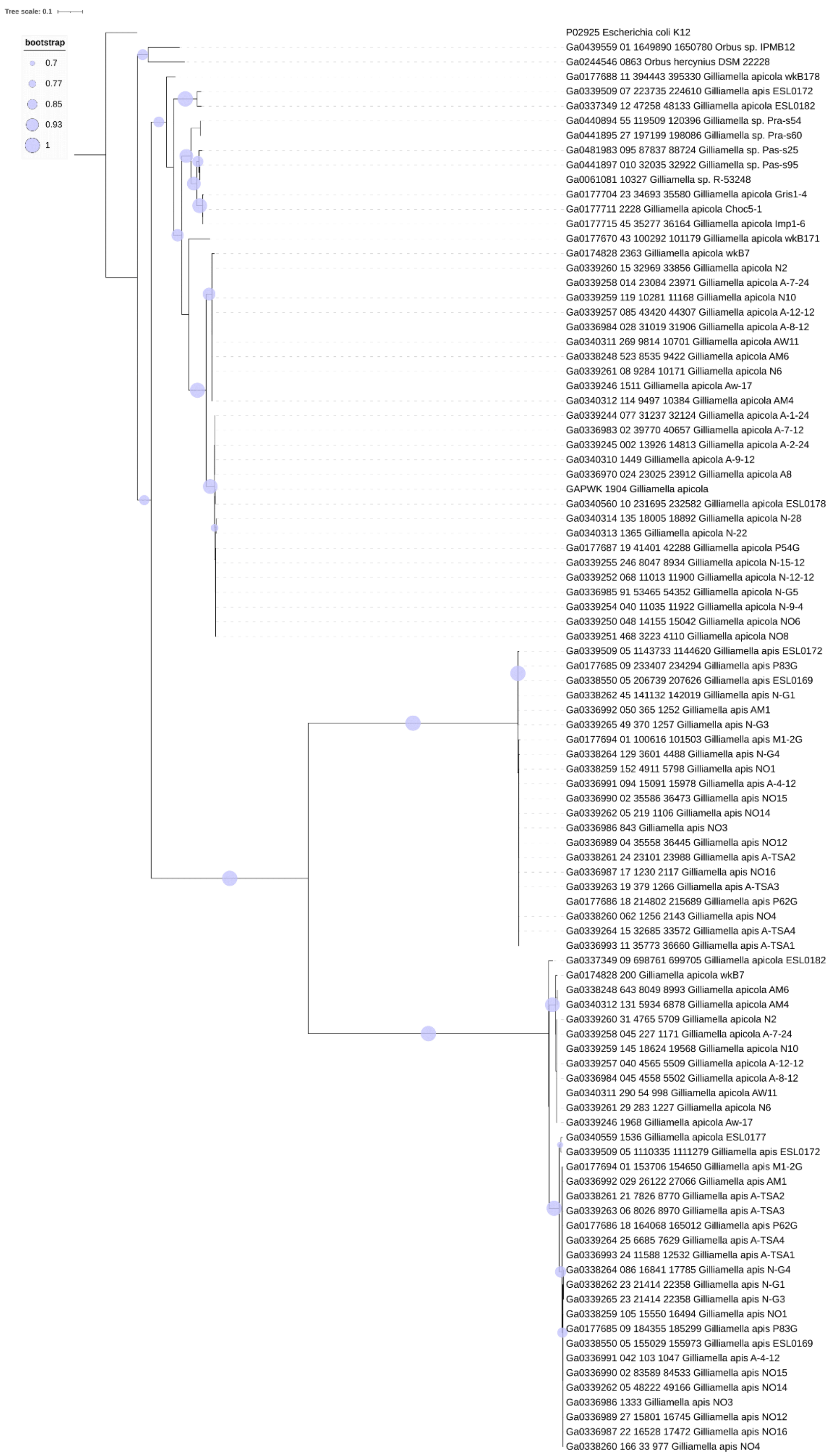
Tree scale: 0.1
bootstrap
0.7
0.77
0.85
0.93
1
P02925 Escherichia coli K12
Ga0439559 01 1649890 1650780 Orbus sp. IPMB12
Ga0244546 0863 Orbus hercynius DSM 22228
Ga0177688 11 394443 395330 Gilliamella apicola wkB178
Ga0339509 07 223735 224610 Gilliamella apis ESL0172
Ga0337349 12 47258 48133 Gilliamella apicola ESL0182
Ga0440894 55 119509 120396 Gilliamella sp. Pra-s54
Ga0441895 27 197199 198086 Gilliamella sp. Pra-s60
Ga0481983 095 87837 88724 Gilliamella sp. Pas-s25
Ga0441897 010 32035 32922 Gilliamella sp. Pas-s95
Ga0061081 10327 Gilliamella sp. R-53248
Ga0177704 23 34693 35580 Gilliamella apicola Gris1-4
Ga0177711 2228 Gilliamella apicola Choc5-1
Ga0177715 45 35277 36164 Gilliamella apicola Imp1-6
Ga0177670 43 100292 101179 Gilliamella apicola wkB171
Ga0174828 2363 Gilliamella apicola wkB7
Ga0339260 15 32969 33856 Gilliamella apicola N2
Ga0339258 014 23084 23971 Gilliamella apicola A-7-24
Ga0339259 119 10281 11168 Gilliamella apicola N10
Ga0339257 085 43420 44307 Gilliamella apicola A-12-12
Ga0336984 028 31019 31906 Gilliamella apicola A-8-12
Ga0340311 269 9814 10701 Gilliamella apicola AW11
Ga0338248 523 8535 9422 Gilliamella apicola AM6
Ga0339261 08 9284 10171 Gilliamella apicola N6
Ga0339246 1511 Gilliamella apicola Aw-17
Ga0340312 114 9497 10384 Gilliamella apicola AM4
Ga0339244 077 31237 32124 Gilliamella apicola A-1-24
Ga0336983 02 39770 40657 Gilliamella apicola A-7-12
Ga0339245 002 13926 14813 Gilliamella apicola A-2-24
Ga0340310 1449 Gilliamella apicola A-9-12
Ga0336970 024 23025 23912 Gilliamella apicola A8
GAPWK 1904 Gilliamella apicola
Ga0340560 10 231695 232582 Gilliamella apicola ESL0178
Ga0340314 135 18005 18892 Gilliamella apicola N-28
Ga0340313 1365 Gilliamella apicola N-22
Ga0177687 19 41401 42288 Gilliamella apicola P54G
Ga0339255 246 8047 8934 Gilliamella apicola N-15-12
Ga0339252 068 11013 11900 Gilliamella apicola N-12-12
Ga0336985 91 53465 54352 Gilliamella apicola N-G5
Ga0339254 040 11035 11922 Gilliamella apicola N-9-4
Ga0339250 048 14155 15042 Gilliamella apicola NO6
Ga0339251 468 3223 4110 Gilliamella apicola NO8
Ga0339509 05 1143733 1144620 Gilliamella apis ESL0172
Ga0177685 09 233407 234294 Gilliamella apis P83G
Ga0338550 05 206739 207626 Gilliamella apis ESL0169
Ga0338262 45 141132 142019 Gilliamella apis N-G1
Ga0336992 050 365 1252 Gilliamella apis AM1
Ga0339265 49 370 1257 Gilliamella apis N-G3
Ga0177694 01 100616 101503 Gilliamella apis M1-2G
Ga0338264 129 3601 4488 Gilliamella apis N-G4
Ga0338259 152 4911 5798 Gilliamella apis NO1
Ga0336991 094 15091 15978 Gilliamella apis A-4-12
Ga0336990 02 35586 36473 Gilliamella apis NO15
Ga0339262 05 219 1106 Gilliamella apis NO14
Ga0336986 843 Gilliamella apis NO3
Ga0336989 04 35558 36445 Gilliamella apis NO12
Ga0338261 24 23101 23988 Gilliamella apis A-TSA2
Ga0336987 17 1230 2117 Gilliamella apis NO16
Ga0339263 19 379 1266 Gilliamella apis A-TSA3
Ga0177686 18 214802 215689 Gilliamella apis P62G
Ga0338260 062 1256 2143 Gilliamella apis NO4
Ga0339264 15 32685 33572 Gilliamella apis A-TSA4
Ga0336993 11 35773 36660 Gilliamella apis A-TSA1
Ga0337349 09 698761 699705 Gilliamella apicola ESL0182
Ga0174828 200 Gilliamella apicola wkB7
Ga0338248 643 8049 8993 Gilliamella apicola AM6
Ga0340312 131 5934 6878 Gilliamella apicola AM4
Ga0339260 31 4765 5709 Gilliamella apicola N2
Ga0339258 045 227 1171 Gilliamella apicola A-7-24
Ga0339259 145 18624 19568 Gilliamella apicola N10
Ga0339257 040 4565 5509 Gilliamella apicola A-12-12
Ga0336984 045 4558 5502 Gilliamella apicola A-8-12
Ga0340311 290 54 998 Gilliamella apicola AW11
Ga0339261 29 283 1227 Gilliamella apicola N6
Ga0339246 1968 Gilliamella apicola Aw-17
Ga0340559 1536 Gilliamella apicola ESL0177
Ga0339509 05 1110335 1111279 Gilliamella apis ESL0172
Ga0177694 01 153706 154650 Gilliamella apis M1-2G
Ga0336992 029 26122 27066 Gilliamella apis AM1
Ga0338261 21 7826 8770 Gilliamella apis A-TSA2
Ga0339263 06 8026 8970 Gilliamella apis A-TSA3
Ga0177686 18 164068 165012 Gilliamella apis P62G
Ga0339264 25 6685 7629 Gilliamella apis A-TSA4
Ga0336993 24 11588 12532 Gilliamella apis A-TSA1
Ga0338264 086 16841 17785 Gilliamella apis N-G4
Ga0338262 23 21414 22358 Gilliamella apis N-G1
Ga0339265 23 21414 22358 Gilliamella apis N-G3
Ga0338259 105 15550 16494 Gilliamella apis NO1
Ga0177685 09 184355 185299 Gilliamella apis P83G
Ga0338550 05 155029 155973 Gilliamella apis ESL0169
Ga0336991 042 103 1047 Gilliamella apis A-4-12
Ga0336990 02 83589 84533 Gilliamella apis NO15
Ga0339262 05 48222 49166 Gilliamella apis NO14
Ga0336986 1333 Gilliamella apis NO3
Ga0336989 27 15801 16745 Gilliamella apis NO12
Ga0336987 22 16528 17472 Gilliamella apis NO16
Ga0338260 166 33 977 Gilliamella apis NO4

(C)

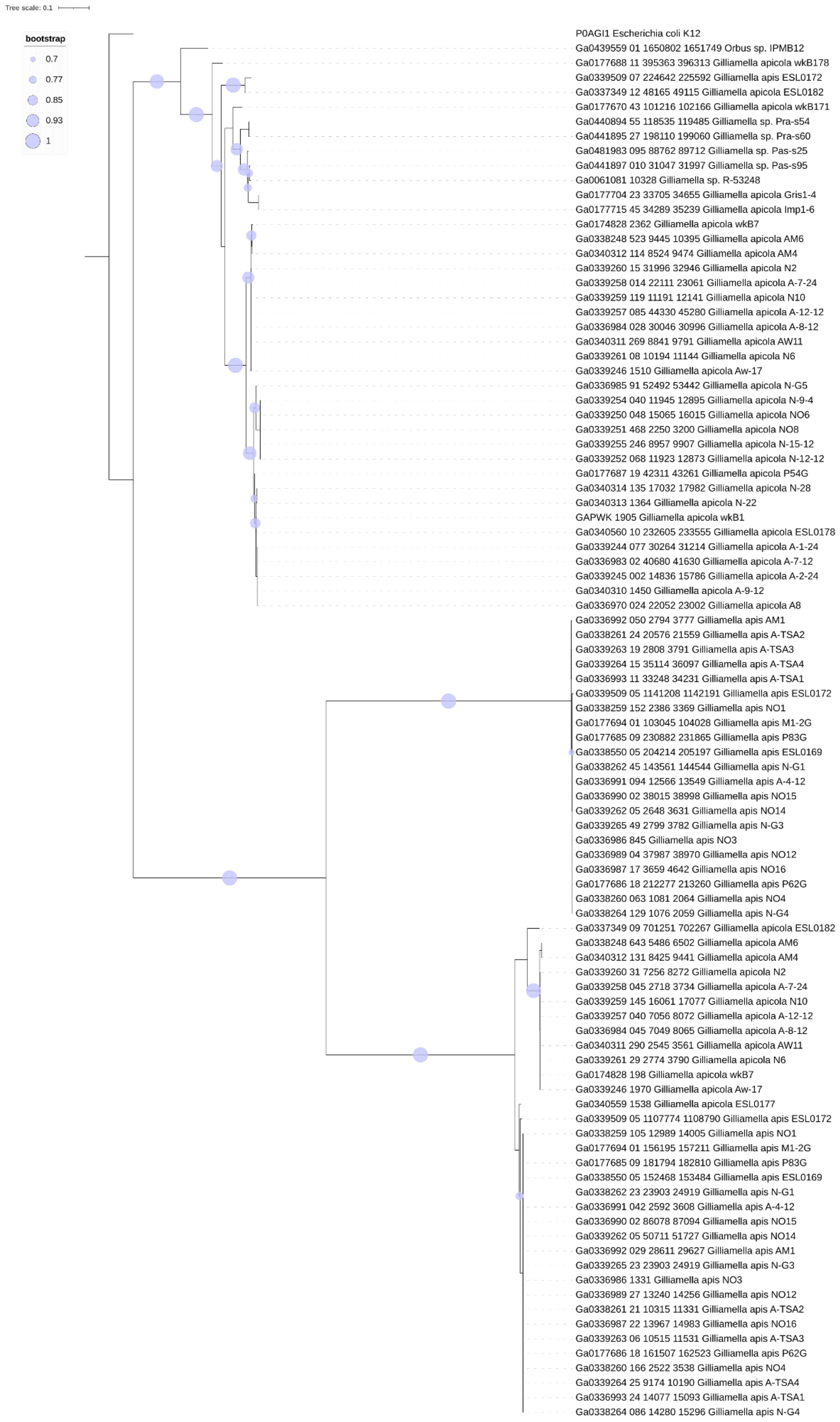
Tree scale: 0.1
bootstrap
0.7
0.77
0.85
0.93
1
P0AGI1 Escherichia coli K12
Ga0439559 01 1650802 1651749 Orbus sp. IPMB12
Ga0177688 11 395363 396313 Gilliamella apicola wkB178
Ga0339509 07 224642 225592 Gilliamella apis ESL0172
Ga0337349 12 48165 49115 Gilliamella apicola ESL0182
Ga0177670 43 101216 102166 Gilliamella apicola wkB171
Ga0440894 55 118535 119485 Gilliamella sp. Pra-s54
Ga0441895 27 198110 199060 Gilliamella sp. Pra-s60
Ga0481983 095 88762 89712 Gilliamella sp. Pas-s25
Ga0441897 010 31047 31997 Gilliamella sp. Pas-s95
Ga0061081 10328 Gilliamella sp. R-53248
Ga0177704 23 33705 34655 Gilliamella apicola Gris1-4
Ga0177715 45 34289 35239 Gilliamella apicola Imp1-6
Ga0174828 2362 Gilliamella apicola wkB7
Ga0338248 523 9445 10395 Gilliamella apicola AM6
Ga0340312 114 8524 9474 Gilliamella apicola AM4
Ga0339260 15 31996 32946 Gilliamella apicola N2
Ga0339258 014 22111 23061 Gilliamella apicola A-7-24
Ga0339259 119 11191 12141 Gilliamella apicola N10
Ga0339257 085 44330 45280 Gilliamella apicola A-12-12
Ga0336984 028 30046 30996 Gilliamella apicola A-8-12
Ga0340311 269 8841 9791 Gilliamella apicola AW11
Ga0339261 08 10194 11144 Gilliamella apicola N6
Ga0339246 1510 Gilliamella apicola Aw-17
Ga0336985 91 52492 53442 Gilliamella apicola N-G5
Ga0339254 040 11945 12895 Gilliamella apicola N-9-4
Ga0339250 048 15065 16015 Gilliamella apicola NO6
Ga0339251 468 2250 3200 Gilliamella apicola NO8
Ga0339255 246 8957 9907 Gilliamella apicola N-15-12
Ga0339252 068 11923 12873 Gilliamella apicola N-12-12
Ga0177687 19 42311 43261 Gilliamella apicola P54G
Ga0340314 135 17032 17982 Gilliamella apicola N-28
Ga0340313 1364 Gilliamella apicola N-22
GAPWK 1905 Gilliamella apicola wkB1
Ga0340560 10 232605 233555 Gilliamella apicola ESL0178
Ga0339244 077 30264 31214 Gilliamella apicola A-1-24
Ga0336983 02 40680 41630 Gilliamella apicola A-7-12
Ga0339245 002 14836 15786 Gilliamella apicola A-2-24
Ga0340310 1450 Gilliamella apicola A-9-12
Ga0336970 024 22052 23002 Gilliamella apicola A8
Ga0336992 050 2794 3777 Gilliamella apis AM1
Ga0338261 24 20576 21559 Gilliamella apis A-TSA2
Ga0339263 19 2808 3791 Gilliamella apis A-TSA3
Ga0339264 15 35114 36097 Gilliamella apis A-TSA4
Ga0336993 11 33248 34231 Gilliamella apis A-TSA1
Ga0339509 05 1141208 1142191 Gilliamella apis ESL0172
Ga0338259 152 2386 3369 Gilliamella apis NO1
Ga0177694 01 103045 104028 Gilliamella apis M1-2G
Ga0177685 09 230882 231865 Gilliamella apis P83G
Ga0338550 05 204214 205197 Gilliamella apis ESL0169
Ga0338262 45 143561 144544 Gilliamella apis N-G1
Ga0336991 094 12566 13549 Gilliamella apis A-4-12
Ga0336990 02 38015 38998 Gilliamella apis NO15
Ga0339262 05 2648 3631 Gilliamella apis NO14
Ga0339265 49 2799 3782 Gilliamella apis N-G3
Ga0336986 845 Gilliamella apis NO3
Ga0336989 04 37987 38970 Gilliamella apis NO12
Ga0336987 17 3659 4642 Gilliamella apis NO16
Ga0177686 18 212277 213260 Gilliamella apis P62G
Ga0338260 063 1081 2064 Gilliamella apis NO4
Ga0338264 129 1076 2059 Gilliamella apis N-G4
Ga0337349 09 701251 702267 Gilliamella apicola ESL0182
Ga0338248 643 5486 6502 Gilliamella apicola AM6
Ga0340312 131 8425 9441 Gilliamella apicola AM4
Ga0339260 31 7256 8272 Gilliamella apicola N2
Ga0339258 045 2718 3734 Gilliamella apicola A-7-24
Ga0339259 145 16061 17077 Gilliamella apicola N10
Ga0339257 040 7056 8072 Gilliamella apicola A-12-12
Ga0336984 045 7049 8065 Gilliamella apicola A-8-12
Ga0340311 290 2545 3561 Gilliamella apicola AW11
Ga0339261 29 2774 3790 Gilliamella apicola N6
Ga0174828 198 Gilliamella apicola wkB7
Ga0339246 1970 Gilliamella apicola Aw-17
Ga0340559 1538 Gilliamella apicola ESL0177
Ga0339509 05 1107774 1108790 Gilliamella apis ESL0172
Ga0338259 105 12989 14005 Gilliamella apis NO1
Ga0177694 01 156195 157211 Gilliamella apis M1-2G
Ga0177685 09 181794 182810 Gilliamella apis P83G
Ga0338550 05 152468 153484 Gilliamella apis ESL0169
Ga0338262 23 23903 24919 Gilliamella apis N-G1
Ga0336991 042 2592 3608 Gilliamella apis A-4-12
Ga0336990 02 86078 87094 Gilliamella apis NO15
Ga0339262 05 50711 51727 Gilliamella apis NO14
Ga0336992 029 28611 29627 Gilliamella apis AM1
Ga0339265 23 23903 24919 Gilliamella apis N-G3
Ga0336986 1331 Gilliamella apis NO3
Ga0336989 27 13240 14256 Gilliamella apis NO12
Ga0338261 21 10315 11331 Gilliamella apis A-TSA2
Ga0336987 22 13967 14983 Gilliamella apis NO16
Ga0339263 06 10515 11531 Gilliamella apis A-TSA3
Ga0177686 18 161507 162523 Gilliamella apis P62G
Ga0338260 166 2522 3538 Gilliamella apis NO4
Ga0339264 25 9174 10190 Gilliamella apis A-TSA4
Ga0336993 24 14077 15093 Gilliamella apis A-TSA1
Ga0338264 086 14280 15296 Gilliamella apis N-G4

(D)

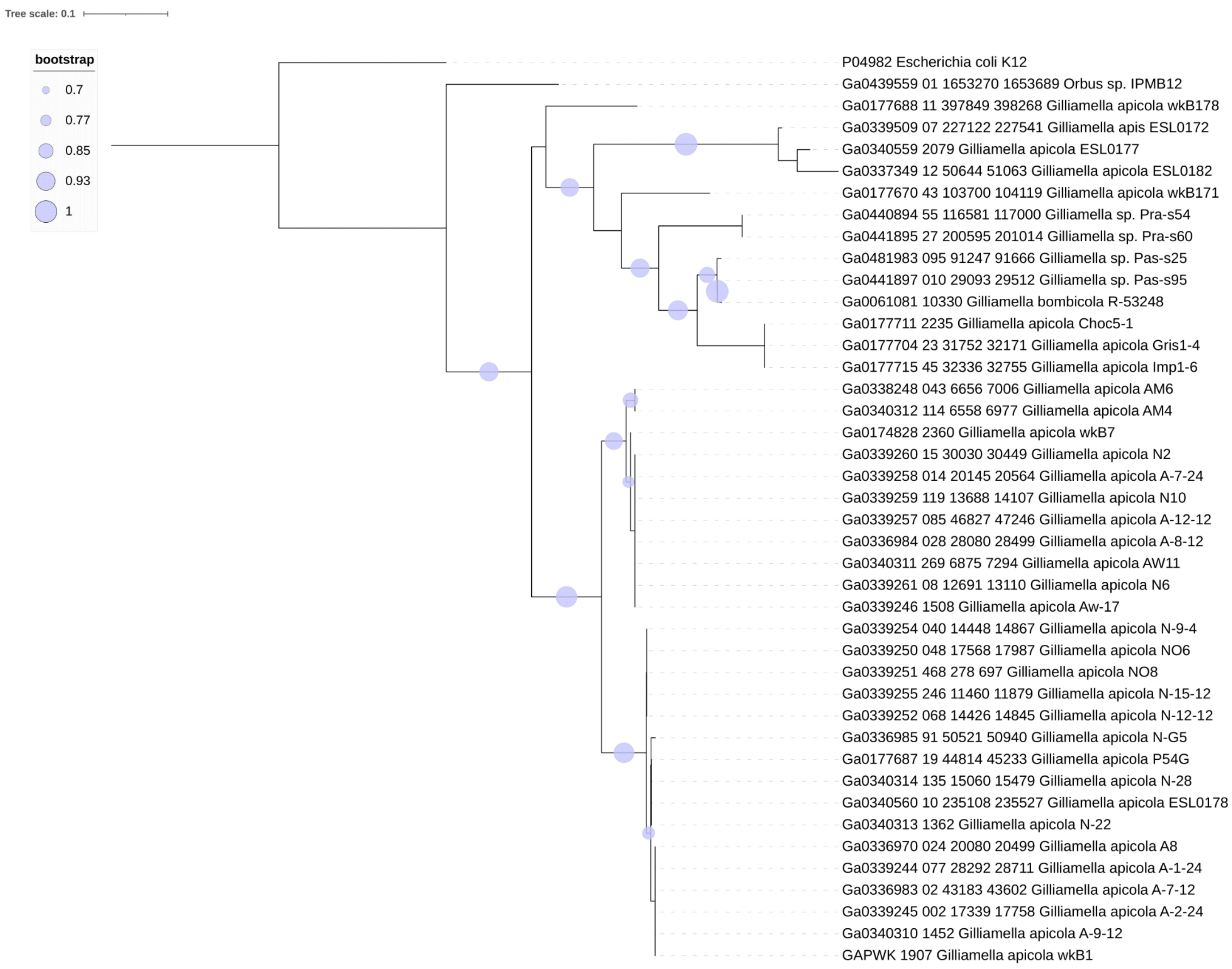

Supplementary Figure S7: Phylogenetic analysis of the *rbsA*, *rbsB*, *rbsC*, and *rbsD* genes found in the 95 *Gilliamella* genomes in this study, along with those in other *Orbaceae* genomes in the IMG/M database, and the *Escherichia coli* K12 *rbs* Swiss-Prot *genes* to form an outgroup (as of August 2nd, 2023). The identifier for the *E. coli rbs* homolog is taken from UniProtKB, while identifiers in all other cases were parsed directly from the IMG/M database. Formatting and annotation was done via iTOL.

References

1. **Praet J, Cnockaert M, Meeus I, Smagghe G, Vandamme P**. Gilliamella intestini sp. nov., Gilliamella bombicola sp. nov., Gilliamella bombi sp. nov. and Gilliamella mensalis sp. nov.: Four novel Gilliamella species isolated from the bumblebee gut. *Syst Appl Microbiol* 2017;40:199–204.
